# Prediction of Rhizoma Drynariae Targets in the Treatment of Osteoarthritis Based on Network Pharmacology and Experimental Verification

**DOI:** 10.1155/2021/5233462

**Published:** 2021-11-18

**Authors:** Guang-yao Chen, Xiao-yu Liu, Jia-qi Chen, Xin-bo Yu, Jing Luo, Ze-ran Yan, Qing-wen Tao

**Affiliations:** ^1^Beijing University of Chinese Medicine, Beijing 100029, China; ^2^Department of TCM Rheumatology, China-Japan Friendship Hospital, Beijing 100029, China; ^3^Beijing Key Lab for Immune-Mediated Inflammatory Diseases, China-Japan Friendship Hospital, Beijing 100029, China

## Abstract

Rhizoma Drynariae has been widely used for the treatment of osteoarthritis (OA), but its potential targets and molecular mechanisms remain to be further explored. Targets of Rhizoma Drynariae and OA were predicted by relevant databases, and a protein-protein interaction (PPI) network was constructed to identify key targets. The Kyoto Encyclopedia of Genes and Genomes (KEGG) enrichment analysis was performed to obtain related pathways and then select significant pathways associated with OA. The OA chondrocyte model was established by inflammatory factor-induced SW1353 chondrocytes, and molecular docking was conducted to verify the above theoretical prediction. The results showed that a total of 86 Rhizoma Drynariae-OA interaction targets were identified, among which IL-6 and AKT1 were the key targets in the PPI network. Luteolin was the most critical component of Rhizoma Drynariae. KEGG results indicated that the effects of Rhizoma Drynariae on OA are associated with the PI3K/AKT, TNF, IL-17, apoptosis, and HIF-1 signaling pathway. The PI3K/AKT pathway can activate the downstream NF-*κ*B pathway and further regulate the transcription and expression of downstream IL-6, IL-17, HIF-1*α*, Bax, and TNF, suggesting that the PI3K/AKT/NF-*κ*B pathway is the critical pathway in the treatment of OA with Rhizoma Drynariae. Active components of Rhizoma Drynariae and key proteins of the PI3K/AKT/NF-*κ*B signaling pathway were subjected to molecular docking, whose results showed that luteolin and IKK-*α* played a critical role. In vitro experiments indicated that both aqueous extracts of Rhizoma Drynariae (AERD) and luteolin inhibited the expression of IL-6 and HIF-1*α* and suppressed the activation of PI3K/AKT/NF-*κ*B, IL-17, and TNF pathways. The measurement of mitochondrial membrane potential (Δ*ψ*m) indicated that AERD and luteolin can decrease the LPS-induced early apoptotic cells. Luteolin had a more prominent inhibitory effect than AERD in the abovementioned in vitro experiments. In conclusion, the therapeutic mechanism of Rhizoma Drynariae against OA may be closely related to the inhibition of the PI3K/AKT/NF-*κ*B pathway and downstream pathways, and luteolin plays a vital role in the treatment.

## 1. Introduction

Osteoarthritis (OA) is the most common joint degenerative disease and the leading cause of disability in elderly people [1]. An imaging study showed that OA affected more than 50% of elderly individuals [2]. Nonsteroidal anti-inflammatory drugs (NSAIDs) are often prescribed to relieve pain and inflammation that result from OA, but their long-term use may lead to serious gastrointestinal side effects [3]. Glucosamine and chondroitin sulfate show significant chondroprotective effects in in vitro experiments; however, its clinical benefits in OA remain controversial [4, 5]. As there is no effective and safe pharmacotherapy for treating OA, the search for disease-modifying osteoarthritis drugs (DMOADs) from natural sources has received much attention [6]. Rhizoma Drynariae is the root of *Drymotaenium fortunei* (Kze.) J. Smith and has been used in traditional Chinese medicine for a long time to treat rheumatic diseases [7]. Rhizoma Drynariae is the principal drug in a vast number of TCM prescriptions that have been recognized as effective in treating OA [8]. However, the specific mechanism of Rhizoma Drynariae in treating OA remains to be further investigated.

**Figure 1:**
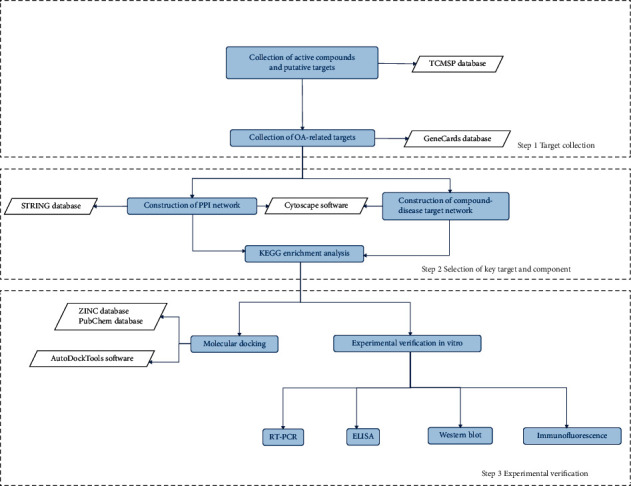
Flowchart of the network pharmacology analysis and experimental validation in this study.

Network pharmacology aims to identify biological networks and analyze the links among drugs, targets, and diseases in the networks [9]. Network pharmacology assists researchers to identify the key components of complex natural drug ingredients and explore their potential therapeutic mechanisms [10]. In this study, network pharmacology was used to predict the key targets of Rhizoma Drynariae that play an important role in OA, and the Kyoto Encyclopedia of Genes and Genomes (KEGG) pathway enrichment was used to predict the critical mechanisms. Subsequently, these mechanisms were ultimately verified through in vitro experiments and molecular docking. A brief flowchart of the method is shown in Figure 1.

## 2. Materials and Methods

### 2.1. Collection of Active Compounds and Putative Targets

The compounds in Rhizoma Drynariae were identified from the traditional Chinese medicines for systems pharmacology database and analysis platform (TCMSP, http://lsp.nwu.edu.cn/tcmsp.php). Oral bioavailability (OB) and drug-likeness (DL) scores were set as the parameters for screening the active compounds, and OB ≥30% and OL ≥0.18 were the criteria for the next step. All the putative targets in Rhizoma Drynariae were also identified by the TCMSP database, and targets related to the active components of Rhizoma Drynariae were chosen for further research.

### 2.2. Collection of OA-Related Targets

The GeneCards database (https://www.genecards.org/), a comprehensive database of human genes, was used for collecting OA-related targets. All the related genes were identified by using “osteoarthritis” as the keyword. The intersecting genes of OA and Rhizoma Drynariae were plotted as a Venn diagram, which was reserved for further study.

### 2.3. Construction of the Protein-Protein Interaction (PPI) Network

To explore the interaction network of the target proteins and further identify the core regulatory targets, the common targets between OA and Rhizoma Drynariae were analyzed by the PPI network using the STRING database (https://string-db.org/Version 11.0). The parameters were set as follows: organism: *Homo sapiens*; active interaction sources: text mining, experiments, databases, coexpression, neighborhood, gene fusion, and co-occurrence. Only the interaction score that reached greater than 0.7 was further analyzed. Cytoscape software (version 3.7.1) was used for network visualization, and a bar graph was generated to show the top 30 genes that had the maximum number of connected genes on the network.

### 2.4. Construction of the Compound-Target Network

Cytoscape software was used to visualize and analyze molecular interactions. The active compounds and intersection genes of Rhizoma Drynariae and OA were loaded into Cytoscape to construct the compound-target network for subsequent analysis.

### 2.5. KEGG Enrichment Analysis

KEGG was used to analyze which signaling pathways the intersection genes of Rhizoma Drynariae and OA were associated within this study. The clusterProfiler package in R Studio was used for KEGG analysis. Pathways satisfying *p* < 0.05 were considered as significant. The significant pathways were further selected to identify critical pathways associated with OA in accordance with previous literature.

### 2.6. Molecular Docking of Active Compound and Key Target

Molecular docking is a program for demonstrating the binding affinities between ligands and the active sites of target proteins. In this study, the molecular structures of the ligand and the target protein were downloaded from the ZINC database (https://zinc.docking.org/) and PubChem database (https://pubchem.ncbi.nlm.nih.gov/), respectively. All receptors and ligands were prepared using AutoDockTools (version 1.5.6). The three-dimensional structures of ligands were imported into AutoDockTools to identify their rotatable bonds and then saved in PDB format. The binding sites on all receptors were defined and saved in GPF format. Finally, docking simulations were conducted via Autodock Vina to generate the docking energy. The heat map was generated according to docking energy. The docked complexes were visualized with PyMOL software.

### 2.7. Experimental Verification In Vitro

#### 2.7.1. Reagents and Antibodies

The reagents and antibodies used in this study included the following: SYBR Green real-time PCR master mix (QPK-201, Toyobo, Japan), 0.25% trypsin-EDTA (Gibco, USA), penicillin-streptomycin (Gibco, USA), fetal bovine serum (ScienCell, USA), Leibovitz's L-15 medium (Gibco, USA), interleukin-1*β* (IL-*1β*) (Peprotech, USA), lipopolysaccharide (LPS) (Sigma, USA), interleukin-17A (IL-17A) (Peprotech, USA), tumor necrosis factor alpha (TNF-*α*) (Peprotech, USA), phosphate-buffered saline (PBS, HyClone, USA), MTS (Promega, USA), RIPA lysis buffer (Solarbio Life Sciences, China), phenylmethanesulfonyl fluoride (PMSF) (Solarbio Life Sciences, China), IL-6 enzyme-linked immunosorbent assay (ELISA) kits (Beijing 4A Biotech, China), polymerase chain reaction (PCR) primers for human IL-6 and *β*-actin (Tingke, China, primer sequences are listed in Table 1), AKT antibody (ab8805, Abcam, UK), p-AKT (S473) antibody (4060S, Cell Signaling, USA), NF-*κ*B p65 antibody (8242T, Cell Signaling, USA), I*κ*B-*α* antibody (4814T, Cell Signaling, USA), Lamin B1 antibody (YT5108, Immunoway, USA), *β*-Actin antibody (TA-09, Zhongshan Jingqiao Biotechnology, China), horseradish peroxidase (HRP) conjugated goat anti-mouse IgG (ZB-5305, Zhongshan Jingqiao Biotechnology, China), HRP-conjugated goat anti-rabbit IgG (ZB-2301, Zhongshan Jingqiao Biotechnology, China), and Alexa Fluor 488-conjugated goat anti-rabbit IgG (H + *L*) (ZF-0511, Zhongshan Jingqiao Biotechnology, China). The following kits were used: nuclear extraction kit (SN0020, Solarbio Life Sciences, China) and mitochondrial membrane potential detection JC-1 kit (551302, BD, USA).

#### 2.7.2. Preparation of Drugs

The aqueous extract of Rhizoma Drynariae (AERD) was purchased from Shanghai Yuanye Bio-Technology Co., Ltd, China. AERD extraction was prepared based on the following protocol: the dried root of *Drymotaenium fortunei* (Kze.) J. Smith was cut into small pieces, decocted with distilled water three times, and then filtered. The filtered solution was concentrated and vacuum-dried to obtain AERD. Luteolin was purchased from Chengdu Herbpurify Co., Ltd, China. AERD was dissolved in PBS, and luteolin was dissolved in DMSO. Syringe filters (0.22 *μ*m) were used to filter the drug solutions to ensure sterility before use.

#### 2.7.3. Liquid Chromatography-Mass Spectrometry

The active compounds of AERD selected from TCMSP were identified by liquid chromatography-mass spectrometry (LC-MS). 20 mg of AERD was dissolved with 1 mL of deionized water and sonicated for 30 min. After centrifugation (12600 g/min, 10 min), the supernatant was filtered with a microporous membrane (0.45 *μ*m). The sample was then measured by ultrahigh performance liquid chromatography (UHPLC) (Shimadzu, Japan) coupled with a SCIEX tripleTOF 5600+ mass spectrometer. The results were compared with the MassBank online Spectral Database (https://massbank.eu/MassBank/), ReSpect DB (http://spectra.psc.riken.jp), and GNPS platform (https://gnps.ucsd.edu/).

#### 2.7.4. Experimental Validation

The specific methods of the validation experiments, including cell culture, MTS assay, RNA isolation, PCR, ELISA, western blotting, and immunofluorescence, are shown in our previous research [11]. The mitochondrial membrane potential (Δ*ψ*m) was measured by flow cytometry (BD FACSCanto II, USA) using the mitochondrial membrane potential detection JC-1 kit according to the manufacturer's instructions.

#### 2.7.5. Statistical Analysis

Continuous variables are presented as mean ± standard deviation (SDs). Student's *t*-test was used to evaluate the differences between the two groups, and *p* < 0.05 was considered to indicate a statistically significant difference.

## 3. Results

### 3.1. Active Compounds and Targets of Rhizoma Drynariae

**Figure 2:**
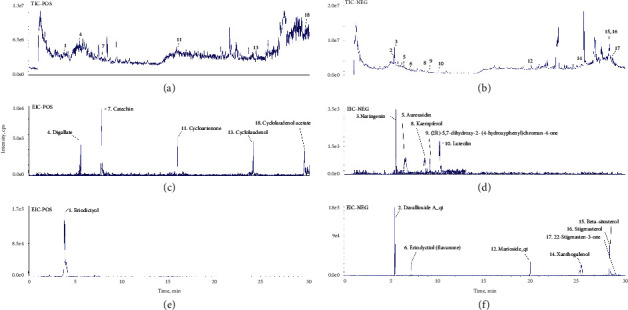
Total ion chromatography (TIC) on positive (a) and negative (b) and extraction ion chromatography (EIC, C-F) of the aqueous extract of Rhizoma Drynariae (AERD).

A total of 71 active compounds were identified in Rhizoma Drynariae by the TCMSP database, among which 18 satisfied the criteria of OB ≥30% and OL ≥0.18, and the results of LC-MS showed that all the 18 active components were identified in AERD (Figure 2), and the chemical identification of each component was shown in Table 2. A total of 139 putative targets corresponding to the 18 active compounds were retrieved from the TCMSP database (Supplementary Table 1).

### 3.2. OA-Related Targets

**Figure 3:**
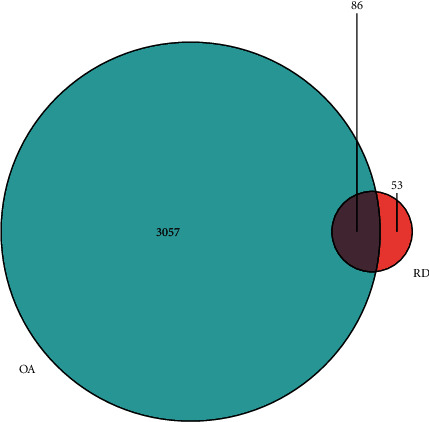
The number of OA-related targets, Rhizoma Drynariae-related targets, and the interaction targets are shown in the Venn diagram.

A total of 3143 OA-related targets were identified in the GeneCards database, of which 86 targets were associated with Rhizoma Drynariae. Detailed information on OA-related targets was provided in Supplementary Table 2. The Venn diagram was generated to demonstrate the number of Rhizoma Drynariae-OA interaction targets (Figure 3).

### 3.3. PPI Network

**Figure 4:**
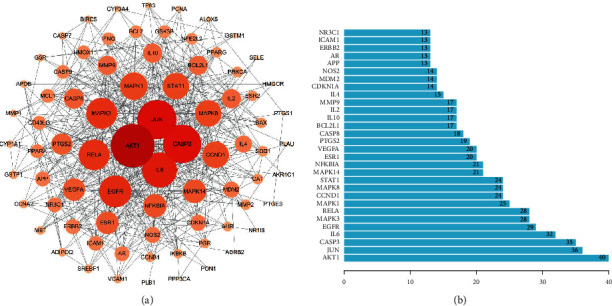
(a) The PPI network of the 86 Rhizoma Drynariae-OA interaction targets. The size indicates the number of connections; the larger the number is, the more connections there are in the network. (b) Barplot of the top 30 Rhizoma Drynariae-OA interaction targets, sorted by target connectivity from large to small in the PPI network.

The interactions between the 86 Rhizoma Drynariae-OA interaction targets were used to construct the PPI network and visualized by Cytoscape (Figure 4(a)). The results showed that AKT1, JUN, CASP3, and IL-6 had the most relative connections with other genes, indicating that they were the most critical gene targets in this network. IL-6 is a vital end product of multiple inflammatory signaling pathways, and therefore, it is selected as an indicator to determine the curative efficacy of the drugs. Other hub genes included JUN, CASP3, MAPK3, PTGS2, EGFR, VEGFA, MMP9, RELA, and BCL2L1 (Figure 4(b)).

### 3.4. Compound-Target Network

**Figure 5:**
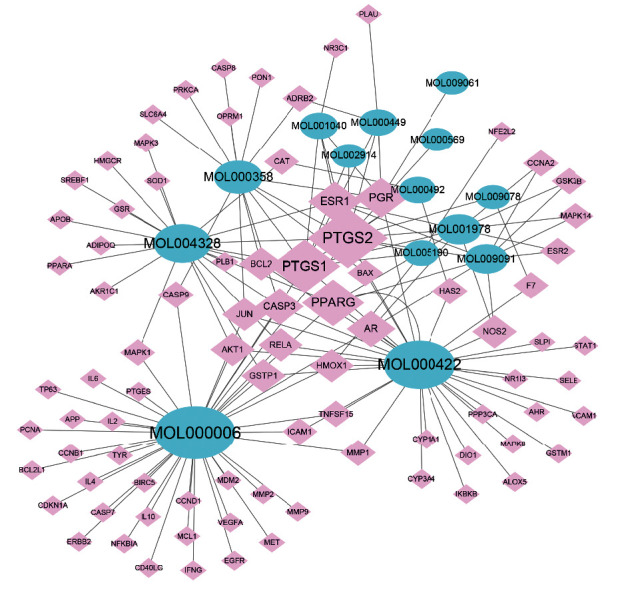
The compound-target network of active compounds and Rhizoma Drynariae-OA interaction targets. The blue nodes represent active compounds of Rhizoma Drynariae; the pink nodes represent Rhizoma Drynariae-OA interaction genes. The larger the node is, the more connections the gene or active compound has.

The compound-target network was built and visualized in accordance with active compounds and Rhizoma Drynariae-OA interaction targets (Figure 5). The results suggested that 14 of the 18 active compounds, including luteolin, kaempferol, naringenin, beta-sitosterol, and aureusidin, were involved in the network. Among the abovementioned, luteolin had a maximum of 41 connections with Rhizoma Drynariae-OA interaction targets, suggesting that luteolin played a central role in the effects of Rhizoma Drynariae in the treatment of OA.

### 3.5. KEGG Enrichment Analysis

**Figure 6:**
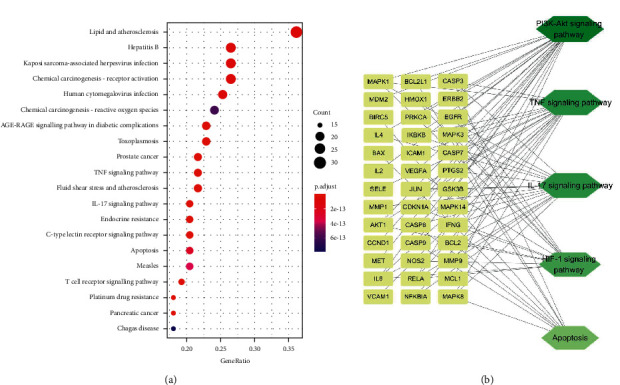
(a) Dot plot for the top 20 KEGG pathways. The size of nodes represents enriched counts. The colors represent significant *p*values (*p* < 0.05); red represents low *p*values, and blue represents high *p*values. (b) A network of 5 OA-related pathways identified from KEGG enrichment analysis and their corresponding targets. Hexagons represent pathways, and rectangles represent the corresponding genes.

86 Rhizoma Drynariae-OA interaction targets were subjected to the KEGG enrichment analysis. Pathways satisfying *p* < 0.05 were considered statistically important, and the top 20 pathways were visualized by a dot plot (Figure 6). The combination of the KEGG enrichment results and systematical literature identified the OA-related pathways, including the PI3K/AKT pathway [12], TNF pathway [13], IL-17 pathway [14], apoptosis pathway [15], and HIF-1 pathway [16]. The PI3K/AKT pathway has the maximum connections with Rhizoma Drynariae-OA interaction targets, and it can also activate the NF-*κ*B pathway to enhance the transcription of TNF-*α*, IL-17, Bax, and HIF-1*α* and thereby regulate the activation of the TNF pathway, IL-17 pathway, apoptosis pathway, and HIF-1 pathway [17–20]. Therefore, the PI3K/AKT/NF-*κ*B pathway was selected for further experimental verification and molecular docking.

### 3.6. Molecular Docking of Active Compound and Key Target

**Figure 7:**
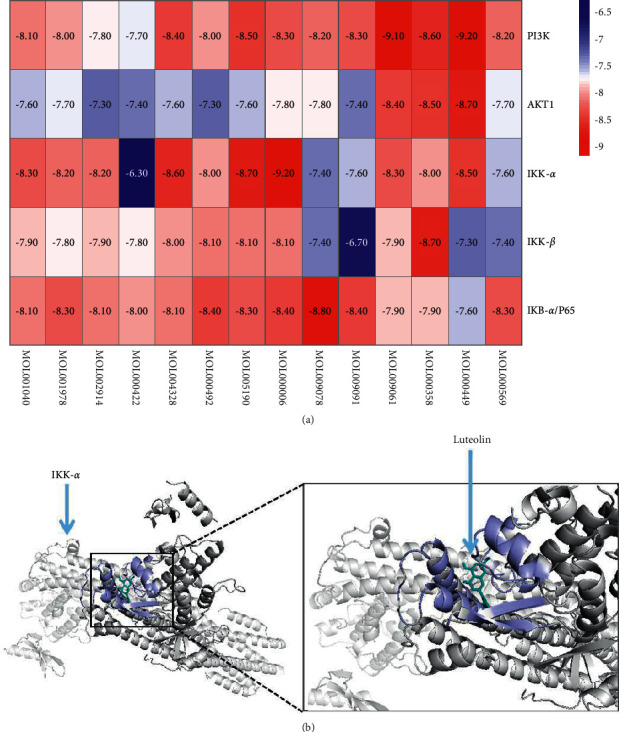
(a) The heat map of binding energy between the active compounds involved in the C-T network and PI3K, AKT1, IKK-*α*, IKK-*β*, and IKB-*α*/p65. (b) Molecular docking site of IKK-*α* and luteolin.

Molecular docking was conducted to predict the binding energy between the active compounds involved in the C-T network and key proteins of the PI3K/AKT/NF-*κ*B pathway (PI3K, AKT1, IKK-*α*, IKK-*β*, and IKB-*α*/p65), whose results were visualized by a heat map (Figure 7(a)). Luteolin has larger binding energy with key proteins relative to the rest of the active compounds, which confirms that luteolin may be a key compound in the treatment of OA with Rhizoma Drynariae. Luteolin has the maximum binding energy with IKK-*α* (−9.20 kcal/mol), which may play a significant role in the effect of luteolin on the PI3K/AKT/NF-*κ*B pathway. The docking site of luteolin and IKK-*α* is visualized in Figure 7(b).

### 3.7. Effects of AERD and Luteolin on SW1353 Cell Viability

**Figure 8:**
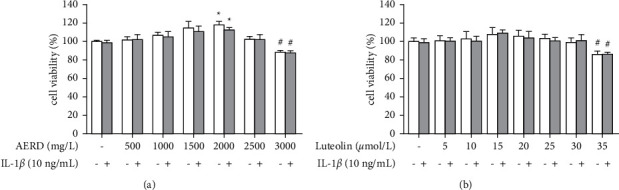
The viability of SW1353 cells after treatment with different concentrations of the aqueous extract of Rhizoma Drynariae (AERD) or luteolin, with or without 10 ng/mL IL-1*β*. (a) SW1353 cells were treated with different concentrations of AERD for 12 h with or without 10 ng/mL IL-1*β*. (b) SW1353 cells were treated with different concentrations of luteolin for 12 h with or without 10 ng/mL IL-1*β*. The data are derived from three independent experiments and are expressed as mean ± standard deviation (^*∗*^*p* < 0.05: increase compared with the control group; *p* < 0.05: decrease compared with the control group).

To determine the appropriate treatment concentration for SW1353 cells, the cells were treated with different concentrations of AERD or luteolin for 12 h, with or without 10 ng/mL IL-1*β*. The MTS assay results indicated that compared with that of the control group, 2000 mg/L AERD promoted cell viability, 2500 mg/L AERD exhibited no effect on cell viability, and 3000 mg/L AERD significantly inhibited cell proliferation. Luteolin had no significant effect on cell proliferation at concentrations of 5 *μ*mol/L, 10 *μ*mol/L, 15 *μ*mol/L, 20 *μ*mol/L, 25 *μ*mol/L, and 30 *μ*mol/L, but cell viability was inhibited at a concentration of 35 *µ*mol/L (Figure 8). Treatment with 10 ng/mL IL-1*β* had no effect on cell viability with different concentrations of AERD and luteolin. Therefore, 2500 mg/L AERD and 30 *μ*mol/L luteolin were selected as the maximum intervention concentrations of SW1353 cells.

### 3.8. Effects of AERD and Luteolin on IL-6 Expression in IL-1*β*-Treated SW1353 Cells

**Figure 9:**
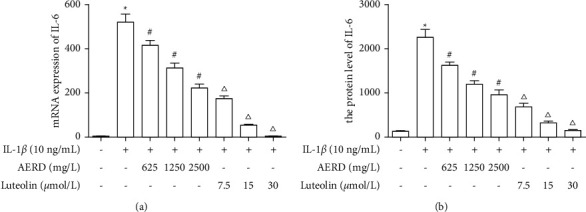
mRNA and protein levels of IL-6 in IL-1*β*-treated SW1353 cells pretreated with the aqueous extract of Rhizoma Drynariae (AERD) or luteolin. SW1353 cells were pretreated with 625 mg/L, 1250 mg/L, or 2500 mg/L AERD or 7.5 *μ*mol/L, 15 *μ*mol/L, or 30 *μ*mol/L luteolin for 1 h, and then treated with 10 ng/mL IL-1*β* for 12 h. (a) mRNA expression of IL-6 in IL-1*β*-treated SW1353 cells pretreated with AERD or luteolin. (b) The protein levels of IL-6 in IL-1*β*-treated SW1353 cells pretreated with AERD or luteolin. The data are derived from three independent experiments and expressed as the mean ± standard deviation (^*∗*^*p* < 0.05: increase significantly compared with the control group; *p* < 0.05: decrease with the concentration of AERD compared with the IL-1*β*-treated group; and *p* < 0.05: significance differences between low dose, medium dose, and high dose of luteolin and AERD).

To detect the effects of AERD and luteolin on IL-6 expression in IL-1*β*-treated SW1353 cells, SW1353 cells were pretreated with 625 mg/L, 1250 mg/L, or 2500 mg/L AERD or 7.5 *μ*mol/L, 15 *μ*mol/L, or 30 *μ*mol/L luteolin for 1 h, and then treated with 10 ng/mL IL-1*β* for 12 h. RT-PCR was used to detect mRNA expression, and ELISA was used to detect the protein levels in the supernatant. RT-PCR results indicated that the mRNA expression of SW1353 cells was significantly increased after stimulation with IL-1*β* (upregulated approximately 500-fold). Both AERD and luteolin inhibited mRNA expression and showed dose-dependent effects. However, the highest concentration of luteolin, which can inhibit IL-6 mRNA expression to nearly normal levels (2-fold), was significantly more effective than AERD. The results of ELISA detection of the protein levels in supernatant were consistent with the trends observed by PCR. The results suggested that IL-1*β* promoted the transcription of IL-6 in SW1353 cells and led to the increased expression of IL-6 protein. This response was reversed by AERD and luteolin, and luteolin may be the key component of Rhizoma Drynariae in the treatment of OA (Figure 9).

### 3.9. Effects of AERD and Luteolin on IL-1*β*-Induced Activation of the PI3K/AKT Pathway

**Figure 10:**
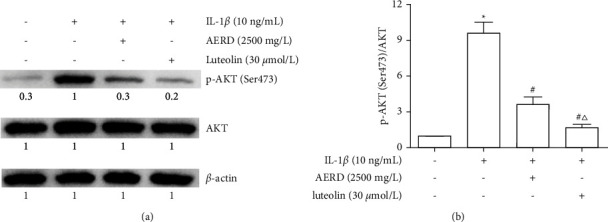
p-AKT levels in IL-1*β*-treated SW1353 cells pretreated with the aqueous extract of Rhizoma Drynariae (AERD) or luteolin. SW1353 cells were pretreated with 2500 mg/L AERD or 30 *μ*mol/L luteolin for 1 h and then treated with 10 ng/mL IL-1*β* for 12 h. p-AKT (Ser473)/AKT levels were measured by western blotting. The data are derived from three independent experiments and expressed as mean ± standard deviation (^*∗*^*p* < 0.05: increased when compared with the control group; *p* < 0.05: decreased when compared with the IL-1*β*-treated group; and *p* < 0.05: decreased when compared with the AERD-treated group).

SW1353 cells were pretreated with 2500 mg/L AERD or 30 *μ*mol/L luteolin for 1 h and then stimulated with 10 ng/mL IL-1*β* for 12 h. The results demonstrated that IL-1*β* did not affect the protein expression of AKT but markedly promoted the phosphorylation of AKT (Ser473). Both AERD and luteolin at the highest concentration inhibited p-AKT expression, and the effect of luteolin was more prominent than that of AERD (Figure 10).

### 3.10. Effects of AERD and Luteolin on IL-1*β*-Induced Activation of the NF-*κ*B Pathway

**Figure 11:**
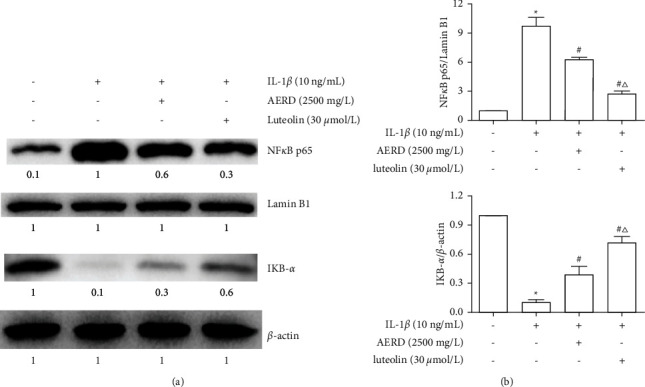
NF-*κ*B p65 and I*κ*B-*α* levels in IL-1*β*-treated SW1353 cells pretreated with the aqueous extract of Rhizoma Drynariae (AERD) and luteolin. SW1353 cells were pretreated with 2500 mg/L AERD or 30 *μ*mol/L luteolin for 1 h and then treated with 10 ng/mL IL-1*β* for 30 min. The I*κ*B-*α* level in the cytoplasm and the NF-*κ*B p65 level in the nucleus were measured by western blotting. Lamin B and *β*-actin were used as internal references for the nuclear and cytoplasmic fractions, respectively. The data are derived from three independent experiments and expressed as the mean ± standard deviation (^*∗*^*p* < 0.05: increased when compared to the control group; ^#^*p* < 0.05: decreased when compared to the IL-1*β-*treated group; and *p* < 0.05: decreased when compared with the AERD-treated group).

The PI3K/AKT signaling pathway can activate the NF-*κ*B signaling pathway by phosphorylating IKK and further lead to IkB-*α* degradation in the cytoplasm and NF-*κ*B nuclear translocation. To further determine the effect of AERD and luteolin intervention on the NF-*κ*B signaling pathways, SW1353 cells were pretreated with 2500 mg/L AERD or 30 *μ*mol/L luteolin for 1 h and then stimulated with 10 ng/mL IL-1*β* for 30 min. A nuclear extraction kit was used to extract the total cytoplasmic and nuclear proteins from the cells. IkB-*α* expression in the cytoplasm and NF-*κ*B P65 expression in the nucleus were determined by western blot, and *β*-actin and Lamin B1 were used as cytoplasmic and nuclear internal references, respectively. After stimulation with IL-1*β*, the NF-*κ*B p65 level in the nuclei was increased, and the IkB-*α* level in the cytoplasm was decreased, which indicated that the NF-*κ*B signaling pathway was activated. These changes could be reversed by pretreatment with AERD and luteolin, and luteolin had a significant effect (Figure 11).

**Figure 12:**
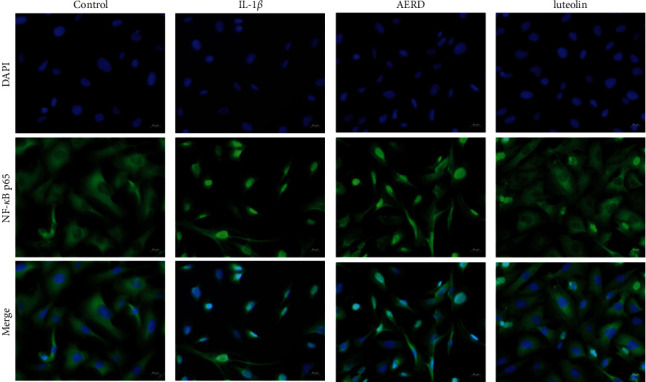
The cells were pretreated with the aqueous extract of Rhizoma Drynariae (AERD) or luteolin for 1 h before IL-1*β* treatment (10 ng/ml). After 30 min of the intervention of IL-1*β*, the localization of NF-*κ*B p65 was visualized with immunofluorescence by fluorescence microscopy after being marked with an anti-NF-*κ*B p65 antibody (green). The cells were also marked with DAPI to visualize the nuclei (blue).

Cellular localization of NF-*κ*B p65 can be determined by an immunofluorescence assay. In this study, NF-*κ*B p65 was significantly transferred from the cytoplasm to the nuclei after being stimulated by 10 ng/mL IL-1*β* for 30 min. Results showed that the pretreatment of AERD or luteolin exhibited inhibitory effects on this change to a certain extent, while luteolin had a more remarkable effect (Figure 12).

### 3.11. Effects of AERD and Luteolin on LPS-Induced Changes in the Mitochondrial Membrane Potential (Δ*ψ*m)

**Figure 13:**
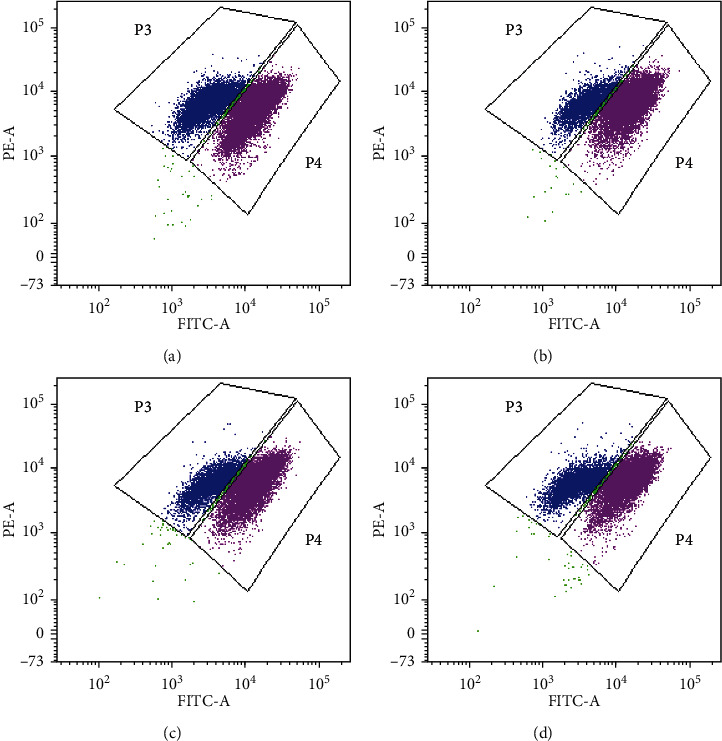
The cells were pretreated with the aqueous extract of Rhizoma Drynariae (AERD) or luteolin for 1 h before LPS stimulation. After 24 h of incubation, flow cytometry was performed to measure the red/green JC-1 fluorescence. P3 represents the green JC-1 fluorescence of cells, and P4 represents the red JC-1 fluorescence of cells. The groups are as follows: (a) the control group; (b) LPS-treated group; (c) AERD-treated group; and (d) luteolin-treated group.

Decreased mitochondrial membrane potential (Δ*ψ*m) is considered as a critical hallmark of early cell apoptosis. The mitochondrial membrane potential (Δ*ψ*m) was measured with the fluorescent mitochondrial probe JC-1. The cells were pretreated with 2500 mg/L AERD or 30 *μ*mol/L luteolin for 1 h and then stimulated with 4 *μ*g/mL LPS for 24 h. Red/green JC-1 fluorescence correlating with the mitochondrial membrane potential (Δ*ψ*m) was measured by flow cytometry (Figure 13). The results of flow cytometric measurement indicated that the stimulation of LPS enhanced the ratio of green JC-1 fluorescence of cells to red JC-1 fluorescence of cells (4.506) compared with that of the control group (1.454), whereas AERD (2.956) and luteolin (2.243) can reverse the changes to a certain extent.

### 3.12. Effects of AERD and Luteolin on the Inhibition of IL-17 and TNF Signaling Pathways

**Figure 14:**
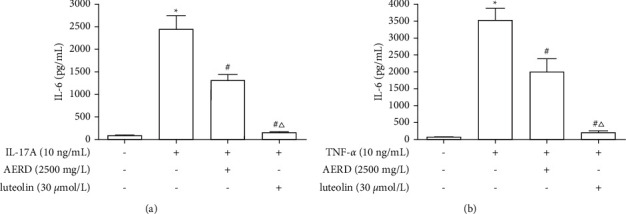
The content of IL-6 in the culture supernatants of IL-17A or TNF-*α* -treated SW1353 cells pretreated with the aqueous extract of Rhizoma Drynariae (AERD) or luteolin. SW1353 cells were pretreated with 2500 mg/L AERD or 30 *μ*mol/L luteolin for 1 h and then treated with 10 ng/mL IL-17A or TNF-*α* for 12h. (a) The content of IL-6 in the culture supernatant of IL-17A-treated SW1353 cells pretreated with AERD or luteolin. (b) The content of IL-6 in the culture supernatant of TNF-*α*-treated SW1353 cells pretreated with AERD or luteolin. The data are derived from three independent experiments and expressed as the mean ± standard deviation (^*∗*^*p* < 0.05: increased when compared to the control group; ^#^*p* < 0.05: decreased when compared to the cytokines-treated group; and ^△^*p* < 0.05: decreased when compared with the AERD-treated group).

10 ng/mL IL-17A was used to activate the IL-17 signaling pathway, and 10 ng/mL TNF-*α* was used to activate the TNF signaling pathway. The SW1353 cells were pretreated with 2500 mg/L AERD or 30 *μ*mol/L luteolin for 1 h and then stimulated with 10 ng/mL IL-17A or TNF-*α* for 12 h. The content of IL-6 in the culture supernatants was determined to reflect the activation extent of the IL-17 and TNF pathways. The results suggested that IL-17A or TNF-*α* can markedly promote the content of IL-6 in the culture supernatants, and the increase of IL-6 was reversed by AERD and luteolin (Figure 14).

### 3.13. Effects of AERD and Luteolin on mRNA Expression of HIF-1*α*

**Figure 15:**
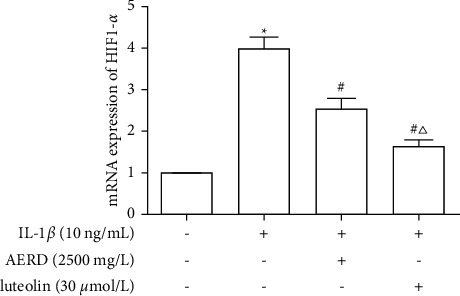
mRNA levels of HIF-1*α* and TNF-*α* in IL-1*β*-treated SW1353 cells pretreated with the aqueous extract of Rhizoma Drynariae (AERD) or luteolin. SW1353 cells were pretreated with 2500 mg/L AERD or 30 *μ*mol/L luteolin for 1 h and then treated with 10 ng/mL IL-1*β* for 12h. (a) mRNA expression of HIF-1*α* in IL-1*β*-treated SW1353 cells pretreated with AERD or luteolin. (b) mRNA expression of TNF-*α* in IL-1*β*-treated SW1353 cells pretreated with AERD or luteolin. The data are derived from three independent experiments and expressed as mean ± standard deviation (^*∗*^*p* < 0.05: increase compared to the control group; ^#^*p* < 0.05: decrease compared to the IL-1*β*-treated group; and ^△^*p* < 0.05: decrease compared with the AERD-treated group).

The SW1353 cells were pretreated with 2500 mg/L AERD or 30 *μ*mol/L luteolin for 1 h and then stimulated with 10 ng/mL IL-1*β* for 12 h. The results indicated that the stimulation of IL-1*β* remarkedly increased mRNA expression of HIF-1*α*; however, AERD and luteolin can inhibit the IL-1*β*-induced mRNA expression of HIF-1*α* and luteolin had a significant effect (Figure 15).

## 4. Discusssion

Network pharmacology was conducted to predict the key targets and therapeutic mechanisms of Rhizoma Drynariae for the treatment of OA, whose results showed that luteolin was the most critical component of Rhizoma Drynariae for OA treatment. KEGG results indicated that the curative effects of Rhizoma Drynariae depend on the PI3K/AKT signaling pathway, TNF pathway, IL-17 pathway, apoptosis pathway, and HIF-1 pathway.

An increasing number of studies have demonstrated that the low-grade inflammation of chondrocytes is closely associated with the progression of OA, suggesting that inhibition of inflammation in OA could be a promising therapeutic strategy [21]. IL-6 was first cloned in the 1980s and verified to promote the activation of T and B lymphocytes [22]. Subsequent studies have shown that IL-6 can be produced by various cells of the human body and has a series of functions, such as modulating the immune system [23], hematopoietic system [24], and neuroendocrine system [25]. IL-6 is produced by synoviocytes and chondrocytes in joints and is considered to be a crucial regulator of cartilage inflammation [26]. Studies have shown that the use of specific IL-6 inhibitors can effectively reduce the inflammatory response in OA model rats, indicating that inhibition of IL-6 is an appealing potential approach in the treatment of OA [27].

In the study, the PPI network results suggested that IL-6 is the most critical target of Rhizoma Drynariae in the treatment of OA. Therefore, the expression of IL-6 at the mRNA and protein level was selected as the basis for the evaluation of the effect of AERD and luteolin on OA. In vitro experiments showed that the pretreatment of AERD or luteolin can exhibit a significant inhibitory effect on IL-6 expression and show a dose-dependent effect. The inhibitory effect of luteolin on IL-6 was more significant than that of AERD, and the maximum concentration of luteolin restored IL-6 to the normal level.

The pathways predicted by KEGG were further analyzed in combination with the literature. The PI3K/AKT signaling pathway is highly correlated with many pathological conditions, such as cell proliferation [28], cell differentiation [29], cell apoptosis [30], cell autophagy [31], and inflammatory response [32]. Numerous studies have confirmed that aberrant activation of the PI3K/AKT signaling pathway has been implicated in tumorigenesis and migration [33], diabetes [34], atherosclerosis [35], and rheumatoid arthritis [36]. After stimulation by related inflammatory factors, PI3K is activated, which is accompanied by molecular conformational changes that trigger the phosphorylation of AKT. Phosphorylated AKT can further lead to the polyubiquitination and proteasomal degradation of I*κ*B-*α* in the cytoplasm, which results in the translocation of NF-*κ*B p65 into the nucleus and promotes the transcription of IL-6, TNF-*α*, IL-17, Bax, and HIF-1*α*. TNF-*α*, IL-17, and HIF-1*α* can activate TNF, IL-17, and HIF pathways, respectively [37, 38]. Bcl2 and Bax can regulate apoptotic pathways [39]. Thus, the PI3K/AKT signaling pathway can modulate TNF, IL-17, and HIF and apoptotic pathways by its regulatory effect on the NF-*κ*B signaling pathway.

It is found that, after IL-1*β* stimulation, the phosphorylated AKT of SW1353 cells decreases in I*κ*B-*α* in the cytoplasm and increases in NF-*κ*B p65 in the nucleus, suggesting the abnormal activation of the PI3K/AKT/NF-*κ*B signaling pathway. Simultaneously, the elevation of HIF-1*α* at the mRNA level suggests that IL-1*β* may activate the HIF-1*α* pathway via promoting the synthesis of HIF-1*α*. After we stimulated the cells with LPS (an inflammatory stimulator which is more intense than IL-1*β*), the mitochondrial membrane potential (Δ*ψ*m) fell, which is the sign of cell early apoptosis [40]. As two significant proinflammatory cytokines, IL-17A and TNF-*α* can activate IL-17 and TNF pathways, respectively, resulting in abundant secretion of inflammatory factors, such as IL-6. The pretreatment of AERD or luteolin can reverse these changes to a certain extent, and luteolin had a more significant effect.

In conclusion, luteolin is the most critical component of Rhizoma Drynariae in the treatment of OA, whose mechanism may be strongly associated with its inhibitory effect on IL-6 expression via the PI3K/AKT/NF-*κ*B pathway and their regulatory role on their downstream signaling pathways. The inadequacy of our study is that the therapeutic effects and mechanism of Rhizoma Drynariae and the key component luteolin on OA were only verified through in vitro experiments. These results should be further validated by in vivo experiments.

## Figures and Tables

**Table 1:** Prime sequences for real-time PCR.

Gene name	Sequence (5′to3′)
Human IL-6 sense	CACTGGTCTTTTGGAGTTTGAG
Human IL-6 antisense	GGACTTTTGTACTCATCTGCAC
Human HIF-1*α* sense	CATCAGCTATTTGCGTGTGAGGA
Human HIF-1*α* antisense	AGCAATTCATCTGTGCTTTCATGTC
Human *β*-actin sense	TGGTGAAGACGCCAGTGGA
Human *β*-actin antisense	GCACCGTCAAGGCTGAGAAC

IL-6, interleukin-6.

**Table 2:** Chemical identification of Drynariae Rhizoma active compounds predicted by TCMSP.

No	RT (min)	Mol. ID	Name	Formula	OB (%)	DL	Ion	Cal. (m/z)	Mea. (m/z)	Error (ppm)	MS/MS
1	3.82	MOL005190	Eriodictyol	C_15_H_12_O_6_	71.79	0.24	M + H	289.0706	289.0711	1.506	289.0711, 185.0035
2	5.39	MOL009078	Davallioside A_qt	C_19_H_19_NO_7_	62.65	0.51	M-H	372.1088	372.1090	3.283	372.1088, 164.0430,120.0548
3	5.57	MOL004328	Naringenin	C_15_H_12_O_5_	59.29	0.21	M-H	271.0611	271.0609	2.95	271.0609,185.0033
4	5.59	MOL000569	Digallate	C_14_H_10_O_9_	61.85	0.26	M + H	323.0397	323.0386	-3.586	323.0386
5	6.60	MOL001978	Aureusidin	C_15_H_10_O_6_	53.42	0.24	M-H	285.0404	285.04	2.23	285.04,224.9613,
6	7.21	MOL002914	Eriodictyol (flavanone)	C_15_H_12_O_2_	41.35	0.24	M-H	223.0764	223.076	2.886	223.076, 109.0276, 57.0392
7	7.91	MOL000492	Catechin	C_15_H_14_O_6_	54.83	0.24	M + H	291.0863	291.0861	-0.737	291.0861, 109.0012
8	8.66	MOL000422	Kaempferol	C_15_H_10_O_6_	41.88	0.24	M-H	285.0404	285.0403	3.282	228.9613,185.0038
9	9.23	MOL001040	(2R)-5,7-dihydroxy-2-(4-hydroxyphenyl)chroman-4-one	C_15_H_12_O_5_	42.36	0.21	M-H	271.0611	271.061	3.32	271.061,185.0026
10	10.26	MOL000006	Luteolin	C_15_H_10_O_6_	36.16	0.25	M-H	285.0404	285.0401	2.58	285.0401,185.0138
11	15.87	MOL009075	Cycloartenone	C_30_H_48_O	40.57	0.79	M + H	425.3778	425.3781	0.722	425.3781
12	20.37	MOL009087	Marioside_qt	C_16_H_24_O_5_	70.79	0.19	M-H	295.1551	295.155	3.387	295.155,164.0452
13	23.99	MOL009076	Cyclolaudenol	C_31_H_52_O	39.05	0.79	M + H	441.409	441.4088	−2.131	441.4088
14	25.34	MOL009091	Xanthogalenol	C_21_H_22_O_5_	41.08	0.32	M-H	353.1394	353.139	1.840	353.1394,191.0571
15	28.62	MOL000358	Beta-sitosterol	C_29_H_50_O	36.91	0.75	M-H	413.3789	413.3782	0.985	413.3782
16	28.62	MOL000449	Stigmasterol	C_29_H_48_O	43.83	0.76	M-H	411.3632	411.3629	1.841	411.3629
17	29.26	MOL009061	22-Stigmasten-3-one	C_29_H_48_O	39.25	0.76	M-H	411.3632	411.363	2.084	411.363
18	29.46	MOL009063	Cyclolaudenol acetate	C_33_H_54_O_2_	41.66	0.79	M + H	483.4197	483.4196	−1.221	483.4196

## Data Availability

The data used to support the findings of this study are available from the corresponding author upon reasonable request.

## Supplementary Materials

Supplementary Materials
*Supplementary Table 1.* Detailed information on putative targets. *Supplementary Table 2.* Detailed information on known OA-related genes.
