# Supplementary material for: Prediction of Rhizoma Drynariae Targets in the Treatment of Osteoarthritis Based on Network Pharmacology and Experimental Verification
Source: Evid Based Complement Alternat Med. 2021 Nov 18;2021:5233462. doi: 10.1155/2021/5233462 (PMC8616695; doi:10.1155/2021/5233462)
Supplement: Supplementary Materials — Supplementary Table 1. Detailed information on putative targets. Supplementary Table 2. Detailed information on known OA-related genes. [file 5233462.f1.zip › 5233462.f1/SUPPLEMENTARY DESCRIPTION.docx]

SUPPLEMENTARY DESCRIPTION

Supplementary Table 1：Detailed information on putative targets

Supplementary Table 2 Detailed information on known OA-related genes
