# Supplementary material for: Prediction of Rhizoma Drynariae Targets in the Treatment of Osteoarthritis Based on Network Pharmacology and Experimental Verification
Source: Evid Based Complement Alternat Med. 2021 Nov 18;2021:5233462. doi: 10.1155/2021/5233462 (PMC8616695; doi:10.1155/2021/5233462)
Supplement: Supplementary Materials — Supplementary Table 1. Detailed information on putative targets. Supplementary Table 2. Detailed information on known OA-related genes. [file 5233462.f1.zip › 5233462.f1/Supplementary Table [1].doc]

Supplementary Table 1：Detailed information on putative targets

| **Molecular name** | **Target** |
| --- | --- |
| (2R)-5,7-dihydroxy-2-(4-hydroxyphenyl)chroman-4-one | PTGS1 |
| (2R)-5,7-dihydroxy-2-(4-hydroxyphenyl)chroman-4-one | ESR1 |
| (2R)-5,7-dihydroxy-2-(4-hydroxyphenyl)chroman-4-one | PTGS2 |
| (2R)-5,7-dihydroxy-2-(4-hydroxyphenyl)chroman-4-one | PGR |
| (2R)-5,7-dihydroxy-2-(4-hydroxyphenyl)chroman-4-one | NR3C2 |
| (2R)-5,7-dihydroxy-2-(4-hydroxyphenyl)chroman-4-one | NR3C1 |
| Aureusidin | NOS2 |
| Aureusidin | PTGS1 |
| Aureusidin | ESR1 |
| Aureusidin | AR |
| Aureusidin | PPARG |
| Aureusidin | PTGS2 |
| Aureusidin | ESR2 |
| Aureusidin | MAPK14 |
| Aureusidin | GSK3B |
| Aureusidin | PRSS1 |
| Aureusidin | CCNA2 |
| Eriodyctiol (flavanone) | PTGS1 |
| Eriodyctiol (flavanone) | PTGS2 |
| Eriodyctiol (flavanone) | NCOA2 |
| Eriodyctiol (flavanone) | PYGM |
| Stigmasterol | PGR |
| Stigmasterol | NR3C2 |
| Stigmasterol | NCOA2 |
| Stigmasterol | ADH1C |
| Stigmasterol | RXRA |
| Stigmasterol | NCOA1 |
| Stigmasterol | PTGS1 |
| Stigmasterol | PTGS2 |
| Stigmasterol | ADRA2A |
| Stigmasterol | SLC6A2 |
| Stigmasterol | SLC6A3 |
| Stigmasterol | ADRB2 |
| Stigmasterol | AKR1B1 |
| Stigmasterol | PLAU |
| Stigmasterol | LTA4H |
| Stigmasterol | MAOB |
| Stigmasterol | MAOA |
| Stigmasterol | CTRB1 |
| Stigmasterol | CHRM3 |
| Stigmasterol | CHRM1 |
| Stigmasterol | ADRB1 |
| Stigmasterol | SCN5A |
| Stigmasterol | ADRA1A |
| Stigmasterol | CHRM2 |
| Stigmasterol | ADRA1B |
| Stigmasterol | GABRA1 |
| beta-sitosterol | PGR |
| beta-sitosterol | NCOA2 |
| beta-sitosterol | PTGS1 |
| beta-sitosterol | PTGS2 |
| beta-sitosterol | KCNH2 |
| beta-sitosterol | CHRM3 |
| beta-sitosterol | CHRM1 |
| beta-sitosterol | SCN5A |
| beta-sitosterol | CHRM4 |
| beta-sitosterol | ADRA1A |
| beta-sitosterol | CHRM2 |
| beta-sitosterol | ADRA1B |
| beta-sitosterol | ADRB2 |
| beta-sitosterol | CHRNA2 |
| beta-sitosterol | SLC6A4 |
| beta-sitosterol | OPRM1 |
| beta-sitosterol | GABRA1 |
| beta-sitosterol | BCL2 |
| beta-sitosterol | BAX |
| beta-sitosterol | CASP9 |
| beta-sitosterol | JUN |
| beta-sitosterol | CASP3 |
| beta-sitosterol | CASP8 |
| beta-sitosterol | PRKCA |
| beta-sitosterol | PON1 |
| beta-sitosterol | MAP2 |
| kaempferol | NOS2 |
| kaempferol | PTGS1 |
| kaempferol | AR |
| kaempferol | PPARG |
| kaempferol | PTGS2 |
| kaempferol | NCOA2 |
| kaempferol | PRSS1 |
| kaempferol | PGR |
| kaempferol | CHRM1 |
| kaempferol | ACHE |
| kaempferol | SLC6A2 |
| kaempferol | CHRM2 |
| kaempferol | ADRA1B |
| kaempferol | GABRA1 |
| kaempferol | F7 |
| kaempferol | RELA |
| kaempferol | IKBKB |
| kaempferol | AKT1 |
| kaempferol | BCL2 |
| kaempferol | BAX |
| kaempferol | TNFSF15 |
| kaempferol | JUN |
| kaempferol | AHSA1 |
| kaempferol | CASP3 |
| kaempferol | MAPK8 |
| kaempferol | MMP1 |
| kaempferol | STAT1 |
| kaempferol | PPARG |
| kaempferol | HMOX1 |
| kaempferol | CYP3A4 |
| kaempferol | CYP1A2 |
| kaempferol | CYP1A1 |
| kaempferol | ICAM1 |
| kaempferol | SELE |
| kaempferol | VCAM1 |
| kaempferol | NR1I2 |
| kaempferol | CYP1B1 |
| kaempferol | ALOX5 |
| kaempferol | HAS2 |
| kaempferol | GSTP1 |
| kaempferol | AHR |
| kaempferol | PSMD3 |
| kaempferol | SLC2A4 |
| kaempferol | NR1I3 |
| kaempferol | INSR |
| kaempferol | DIO1 |
| kaempferol | PPP3CA |
| kaempferol | GSTM1 |
| kaempferol | GSTM2 |
| kaempferol | AKR1C3 |
| kaempferol | SLPI |
| naringenin | PTGS1 |
| naringenin | ESR1 |
| naringenin | PTGS2 |
| naringenin | RELA |
| naringenin | AKT1 |
| naringenin | BCL2 |
| naringenin | MAPK3 |
| naringenin | MAPK1 |
| naringenin | CASP3 |
| naringenin | FASN |
| naringenin | LDLR |
| naringenin | SOD1 |
| naringenin | CAT |
| naringenin | PPARG |
| naringenin | MTTP |
| naringenin | APOB |
| naringenin | PLB1 |
| naringenin | HMGCR |
| naringenin | GSTP1 |
| naringenin | UGT1A1 |
| naringenin | PPARA |
| naringenin | SREBF1 |
| naringenin | GSR |
| naringenin | ABCC1 |
| naringenin | ADIPOQ |
| naringenin | SOAT2 |
| naringenin | AKR1C1 |
| naringenin | GOT1 |
| naringenin | ABAT |
| naringenin | CES1 |
| naringenin | SOAT1 |
| (+)-catechin | PTGS1 |
| (+)-catechin | ESR1 |
| (+)-catechin | PTGS2 |
| (+)-catechin | NCOA2 |
| (+)-catechin | RXRA |
| (+)-catechin | CAT |
| (+)-catechin | HAS2 |
| eriodictyol | PTGS1 |
| eriodictyol | PTGS2 |
| eriodictyol | NCOA2 |
| eriodictyol | HMOX1 |
| eriodictyol | NFE2L2 |
| eriodictyol | NQO1 |
| digallate | PTGS2 |
| digallate | AKR1B1 |
| luteolin | PTGS1 |
| luteolin | AR |
| luteolin | PTGS2 |
| luteolin | PRSS1 |
| luteolin | NCOA2 |
| luteolin | RELA |
| luteolin | EGFR |
| luteolin | AKT1 |
| luteolin | VEGFA |
| luteolin | CCND1 |
| luteolin | BCL2L1 |
| luteolin | CDKN1A |
| luteolin | CASP9 |
| luteolin | MMP2 |
| luteolin | MMP9 |
| luteolin | MAPK1 |
| luteolin | IL10 |
| luteolin | RB1 |
| luteolin | TNFSF15 |
| luteolin | JUN |
| luteolin | IL6 |
| luteolin | CASP3 |
| luteolin | TP63 |
| luteolin | NFKBIA |
| luteolin | TOP1 |
| luteolin | MDM2 |
| luteolin | APP |
| luteolin | MMP1 |
| luteolin | PCNA |
| luteolin | ERBB2 |
| luteolin | PPARG |
| luteolin | HMOX1 |
| luteolin | CASP7 |
| luteolin | ICAM1 |
| luteolin | MCL1 |
| luteolin | BIRC5 |
| luteolin | IL2 |
| luteolin | CCNB1 |
| luteolin | TYR |
| luteolin | IFNG |
| luteolin | IL4 |
| luteolin | TOP2A |
| luteolin | GSTP1 |
| luteolin | SLC2A4 |
| luteolin | INSR |
| luteolin | CD40LG |
| luteolin | PTGES |
| luteolin | NUF2 |
| luteolin | ADCY2 |
| luteolin | MET |
| 22-Stigmasten-3-one | PGR |
| cycloartenone | NR3C2 |
| davallioside A_qt | PTGS2 |
| davallioside A_qt | F7 |
| davallioside A_qt | PYGM |
| xanthogalenol | NOS2 |
| xanthogalenol | ESR1 |
| xanthogalenol | AR |
| xanthogalenol | PPARG |
| xanthogalenol | PTGS2 |
| xanthogalenol | ESR2 |
| xanthogalenol | MAPK14 |
| xanthogalenol | GSK3B |
| xanthogalenol | CCNA2 |
