# Supplementary material for: Prediction of Rhizoma Drynariae Targets in the Treatment of Osteoarthritis Based on Network Pharmacology and Experimental Verification
Source: Evid Based Complement Alternat Med. 2021 Nov 18;2021:5233462. doi: 10.1155/2021/5233462 (PMC8616695; doi:10.1155/2021/5233462)
Supplement: Supplementary Materials — Supplementary Table 1. Detailed information on putative targets. Supplementary Table 2. Detailed information on known OA-related genes. [file 5233462.f1.zip › 5233462.f1/Supplementary Table [2] .doc]

Supplementary Table 2 Detailed information on known OA-related genes

PTGS2

TLR4

TNF

IL6

TGFB1

FTO

HLA-DQB1

ESR1

VEGFA

COL2A1

HIF1A

ADIPOQ

TP53

IL1B

LEP

IL10

CRP

ACE

MMP9

NFKB1

COMP

VDR

CXCL8

HMGB1

CTNNB1

MTHFR

IL17A

PPARG

GDF5

SPP1

CCL2

MMP1

IL1RN

NCOA3

BDNF

CXCL12

MIR146A

SIRT1

HLA-DRB1

AKT1

COMT

MMP2

HFE

CDKN2A

AGER

RELA

MMP3

PTEN

NOS3

ASPN

NFE2L2

SMAD3

MAPK1

RETN

PLA2G4A

CCAL1

HLA-B

SERPINE1

PON1

MTOR

IGF1

BCL2

HMOX1

FAS

CXCR4

IL1A

CD44

ESR2

IL18

XRCC4

OS6

OS4

TNFRSF11B

TLR2

SOD2

EDN1

IL4

GSTT1

ICAM1

MIR21

FRZB

NOTCH1

HLA-A

MAPK14

APOA1

SOX9

MIR34A

DOT1L

NOS2

NLRP3

FOXP3

EZH2

TNFSF11

HLA-G

MIR155

GSK3B

RUNX2

NAMPT

GJA1

JUN

BAX

HSPA5

MMP13

LMNA

CYP1A1

MIF

MRC1

CALCA

TLR9

MAPK3

SP1

RAC1

LGALS3

KDR

TP63

COL1A1

LCN2

APOB

MATN3

TTR

CYP19A1

YAP1

FN1

CHI3L1

ALK

TWIST1

HGF

HDAC1

CCN2

FGF2

GNL3

LEPR

TARDBP

BTNL2

SLC2A1

TCF7L2

FOXO1

BMP2

ADAM12

CCAR2

DKK1

FASLG

FLT1

FOXO3

PLAU

IGF2

CCL5

OPRM1

PBRM1

IGFBP3

TNFRSF1A

TLR3

CSMD1

S100A9

EPAS1

FGFR3

SOX2

FGFR1

BCL2L1

TRAF6

ENG

CLU

CD40

IL33

CRTC1

TGFBR2

TNFAIP3

MEFV

PIK3R1

CNR1

NFKBIA

PRKCD

IL15

GRN

VIM

P2RX7

MMP14

TNFRSF1B

LGALS1

MMP7

MIR145

KL

IL12B

VCAM1

CALM1

HOTAIR

TGM2

TRPV1

MALAT1

HAVCR2

KCNJ11

B2M

ELAVL1

CDC5L

IL6R

ITGB2

PKM

WNT5A

SOST

EGR1

HPSE

MYD88

DNMT3A

CCR2

BCL2L11

H19

F2RL1

NOX4

GHR

IL4R

BMP4

SOCS1

SMAD2

BMP7

NGF

ERG

TIMP2

POSTN

MIR140

S100A8

ADAM17

HDAC2

MGP

IL17F

SHC1

CYBA

HSPD1

CAMK2B

MIR29A

CAT

PGF

AHSG

IL22

IKBKB

PTH

S100A4

OLR1

LRP5

FGF21

ADAMTS4

BGLAP

FOS

CDH2

TIMP3

FKBP5

CX3CL1

PTHLH

POMC

PTPN1

CRYAB

AQP1

CHRNA7

MUTYH

MIR223

ALDH1A2

TGFA

MIR27A

IL23A

MTDH

APLN

ORAI1

BDKRB2

CHGA

CCN1

CTSB

MIR221

HLA-DQA2

MIR210

CHUK

KEAP1

PRKCZ

MIR203A

SAA1

MCF2L

TNC

ITGAM

IRF1

ALOX15

ADAMTS5

IL27

SPHK1

GRIN2B

DEFB4A

IL7

TRAF2

JAG1

CCR7

CXCR3

HTRA1

SFTPD

RACK1

HDAC4

ITLN1

IRF5

COL3A1

TRPV4

XBP1

DIO2

IL1R1

RARRES2

INHBA

IL37

MIR181A1

ACAN

CNR2

PADI4

KPNA2

TNFRSF11A

TREM1

IFRD1

CTSK

MSTN

NEAT1

TCF4

FGF1

CCL20

MIR195

ATF3

MEG3

CRH

MIR31

CXCL13

KCNMA1

MIR30A

TACR1

MIR16-1

POR

CD247

SIRT6

YWHAE

SFTPA1

MIR26A1

CYP27B1

TAC1

ABCC4

ADCYAP1

PDPK1

VIP

PTH1R

TRPA1

SREBF2

SUPT3H

ADIPOR1

MIR22

CD47

LGR5

MIR15A

MIR29B1

LGALS9

PVT1

MIR204

ZFP36

NUCB2

BBC3

KLF5

WNT3A

GAS5

MIR141

IL16

MIR206

SEMA3A

MIR125B1

CTSS

SFTPB

CCL11

CCR6

KHDRBS1

DKK3

CCN4

CCL21

PTGES

MIR199A1

PTGER4

ANPEP

FAP

RHOB

CCL18

TUG1

SHOX

ESRRA

ACTG1

FPR2

EPHB4

MIR149

PGK1

BAG1

MIR142

EFEMP1

CCR3

TRIB3

MIR9-1

GREM1

KLF2

S100A12

TRAF1

LOXL2

PEPD

LILRB1

COG5

IKBKE

PLTP

PTGDS

MIR335

SFTPC

GLB1

EFNB2

COL11A1

MIR139

TGFBR3

MIR130A

DDR2

FHL2

SDC4

TMSB4X

LY96

PLEC

SULF1

HDAC8

TRPV6

KDM6B

SOX11

LTBP1

OPRD1

MIR320A

ROR2

PRG4

HRH4

ACP5

CHAT

CLCN7

MFGE8

IHH

OCRL

MIR92A1

RIPK2

TNFAIP6

MIR107

TPSAB1

MIR24-1

MIR27B

SMURF2

GDF2

CXCL11

TNFSF14

TLR10

GRP

DLL1

TIA1

FUT4

LGALS8

MIR106A

TPI1

MIR138-1

GLT8D1

ACLY

MIR26B

TNKS

GLRX

BAG6

TAB2

DANCR

SLC16A4

MAF

SPHK2

CA1

HDAC7

CALM2

SYVN1

FSTL1

JUND

ADAM15

PARD3B

S100A11

COL10A1

DVL2

PDCD5

MIR33A

SETDB1

MIR98

TRIB1

CEMIP

B4GALT1

ANGPTL2

PRDX5

LAG3

FFAR4

RBFOX1

CCL19

LPAR1

MIR449A

SNHG1

ZC3H12A

COL6A3

USP14

NR1D1

LRG1

MIR590

KLRB1

CTHRC1

MIR199A2

DDAH2

MIR128-1

ELF3

VIPR2

MIR193B

DMP1

ANP32A

CCN6

ADAMTS9

MIR125B2

TRAPPC2

COL11A2

PITX1

SULF2

PCSK6

HHIP

ADAM8

MIR181A2

IL18R1

PADI2

TSC22D3

ACSL4

WWP2

MIR486-1

BMP5

MIR373

SOX6

IL34

COL9A1

DNAH10

KCNIP3

TRIB2

TIMP4

NACA2

COL9A2

MIR30B

CMKLR1

CIRBP

UGDH

SOX5

MIR9-2

HIF3A

DIO3

SLC26A2

MIR455

C1QTNF3

MIR370

FIS1

FIP1L1

IBSP

MIR24-2

NFATC4

MIR16-2

XYLT1

MIR33B

EFEMP2

MAZ

KLF15

VPS4B

FGF18

HBP1

MIR381

COL9A3

MIR361

LGR4

SLC39A8

CCL13

MIR9-3

P4HA1

OGN

ADAMTS7

FOLR2

MIR127

RSPO2

CPEB1

MIR181C

MIR365A

CLEC3B

MIR377

RIPK4

UNC5C

CD53

CSGALNACT1

FZD5

NRCAM

HAPLN1

GLIS3

TMEM167A

SECISBP2

UNC5B

ZFP36L1

MIR382

CDR1-AS

MIR4435-2HG

LRCH1

FBLIM1

MMP17

MFAP4

TGIF2

MIR454

P4HA2

SLC25A27

GRINA

CHST11

PRNCR1

CILP

MIR488

STT3B

DUS4L

MIR671

CORT

MIR940

COL6A4P1

UQCC1

MIR448

GPD1L

LNCRNA-ATB

MIRLET7E

SNHG5

MIR885

USP3

NLRP6

MIR324

MIR337

ADAMTS14

SPON1

MIR95

TUT7

TENT5A

HES5

MIR582

CA10

MIR1236

NCALD

TUFT1

MIR300

ERVK-18

DEFB104A

NME8

UFC1

TREH

SCARNA18

SNORD19

TSBP1-AS1

SYNE3

UCMA

YAF2

ZBED3

PART1

GNAS-AS1

OLIG3

MKX

TRIM38

HSH2D

MIR577

CILP2

XCL2

LOXL1-AS1

MIR105-1

OMD

PRTG

MIR203B

LOC109461476

TM2D3

DEFB104B

CHADL

MELTF-AS1

MCM3AP-AS1

NEWENTRY

VIM2P

COX2

FBN1

TGFB3

TGFB2

TGFBR1

COL5A2

UFSP2

TIMP1

MATN1

PTGS1

ADAM10

DCN

COL5A1

ALB

AEBP1

ANKH

COL1A2

KIF22

PHEX

CREB3L1

KCNK15-AS1

PCGEM1

EXT1

PACERR

HGD

MYLK

ACTA2

MYH11

SMAD4

SMAD6

SLC2A10

PMS2P2

F9

KCNJ5

KCTD13

FMOD

GNAS

TRPS1

CANT1

TYR

DDH2

IFNG

CD36

GBA

EXT2

F8

SCARB2

PTPN22

IL13

CCL3

UBA5

UFM1

DDRGK1

UFL1

UFSP1

C4orf47

CSF1

HPGD

C1S

MIA2

ATP7B

ENPP1

ALPL

LMX1B

ADAMTSL1

IL2

ELN

GNA11

CIITA

AKR1C1

PLOD1

TECTB

IL1RAPL2

SERPINA3

CCND1

MMP8

TRAPPC2B

CASR

THBS1

AIP

OSM

MAPK8

SLC40A1

ZMPSTE24

GPR101

KIF7

ZNF687

ADAMTS3

CD40LG

RELN

CXCL1

GALNS

IL11

SMAP2

P4HB

MEN1

AP3D1

BMP6

OPTC

MPO

EGF

CCL4

CSF2

SOD1

FGF23

FBLN2

CD86

THBS3

INA

TRAPPC3

TRAPPC4

TRAPPC9

TRAPPC6B

CREB3L3

TRAPPC1

TRAPPC10

TRAPPC2L

TRAPPC6A

TMED2

TRAPPC5

TRAPPC8

GPR33

TRAPPC3L

TLR5

CASP3

C1R

HSP90B1

PTPN11

NOD2

CANX

MEPE

MIR150

MIR19A

FOXM1

NLRP1

DSPP

GAPDH

LTA

TNFSF13B

PLAUR

STAT3

SLC22A4

TNXB

MATN4

CTSL

ALOX5

COX5A

ANXA1

BRAF

CDK4

RET

HRAS

IDH1

IDH2

KRAS

NRAS

BRCA2

CPT2

IDS

LIG4

LYZ

SLC11A1

ERCC4

TCIRG1

ARID1B

LTBP2

FBLN1

CD244

TNR

KMT2D

FKBP14

COG4

MICA

HNRNPL

TECTA

P3H1

NFKBIL1

NINL

STRC

CEACAM16

OTOG

H2AC18

MIR132

MIR106B

MIR25

MIR93

MIR32

SPDT

CD14

MMP12

IL17RA

SERPINA1

IDUA

TLR8

CSF1R

LOX

FOSL1

SELL

TGIF1

CTLA4

TLR8-AS1

PIK3CG

LIF

GGT1

SERPINH1

CD80

SCN9A

ELANE

CCR5

CASP8

HLA-DQA1

SFRP4

SRC

STAT4

ADA

ITGB5

IL5

EDEM2

LINC00976

CD28

CASP1

CXCL6

SELE

ANXA5

PRKAR1A

CDH1

ACTB

CD4

CDKN1B

VWF

ARSA

MITF

IKZF1

GPT2

REL

PRODH

HAMP

SLC34A1

FMO1

FAM20C

FBN2

SLC34A3

PIGB

HPS5

ICOSLG

SMYD1

GBP4

MRAP

PDZD11

PALM

PALMD

PRDM10

H3-2

LOC102723692

LOC101448202

LOC113939944

CD163

PRL

CDKN1A

LEPQTL1

PLA2G2A

CYCS

F3

ANGPT1

CSF3

CXCL10

CXCL5

TNFRSF10A

F2

GHRHR

IGF1R

TNFRSF13C

CXCL2

CP

SPARC

IGFBP2

MYC

MET

IL2RA

ATM

VCAN

HAS1

CXCL9

PTK2B

CTSD

CALCR

SERPINC1

TNFSF13

HSPA4

SAA4

IL32

PROCR

NR3C1

PTPRC

TEK

LRRK2

XIAP

KDM1A

CR1

IGFBP1

STEAP4

OLAH

CCR1

NPY

IFNB1

TMPO

ITGB3

IL10RA

HP

IL3

CD38

SDC1

SST

CXCR5

PLOD2

BSG

STAT5B

HSP90AA1

APOH

FCGR3A

PNOC

ADAMTS15

CAST

CA2

OPRK1

MIR675

CD79A

PRTN3

FCGR3B

EP300

CXADR

MC1R

HABP2

IL6ST

IL23R

CHIT1

GPT

FGFR2

BRD4

MIR483

ADRB2

TRAF3

NCAM1

BRD2

NR1I3

TSPO

PREP

TFEB

ZFAS1

SMO

NFKB2

BLK

PRKG1

APOE

DRD2

SNCA

GLA

TFRC

ZEB1

ZEB2

F7

ADCY3

GAA

PKLR

SLC11A2

PSAP

NR4A2

TYRP1

AGA

GALNT3

HPD

ERCC6

GUCY2C

SLC26A3

TAC3

RELB

PSTPIP1

GH1

FBLN5

FMR1

GSTA3

CRYAA

MAFB

SLC6A13

SLC25A6

SLC26A4

SLC26A5

SLC39A13

LAMP1

UGCG

CHST3

PECR

GBA2

CCT3

GLYAT

ATG3

CA3

MFAP5

SLC26A8

SLC6A11

HOXD10

SLC35D1

SLC26A1

SLC38A4

TSHZ1

TMEM43

GGA3

GGCT

BLZF1

ACOT11

HPS6

MTX1

SLC26A6

SRSF7

TTC3

CCL1

LZTS1

SLC7A3

PLEKHA7

SLC26A11

OTULIN

DOCK11

STARD10

SYNJ2BP

FCRL3

SUGCT

PCMTD1

CAGE1

EMP1

ARSH

TAAR9

GGT2

EMSY

OR1L1

KRTAP10-1

MACIR

UCA1

MIR10B

MIR222

GGTLC3

MIR212

MIR103A1

RNF217-AS1

SNORA3A

TMPO-AS1

TGFB2-AS1

TGFB2-OT1

MIR6069

ENSG00000259039

CFLAR

CSK

ATP6V0A2

CRBN

HLA-DPB1

MAEA

SLBP

NSD2

USP50

MMP10

CENPP

LOC101928222

BGN

TNFRSF10B

ITGA4

ENO1

MAP2K7

CTSG

STAT1

CFI

TNFSF10

VTN

DNMT1

SELP

NT5E

CX3CR1

ERBB2

INS

JAM3

IL18BP

NOG

THBD

HSD11B1

PDCD1

LRP1

GPI

PPARA

CARD16

MIR17

THY1

PYCARD

RECK

CASP9

IL2RB

CCK

JAK2

APP

SYK

LPL

GRB2

ITGAL

GHRL

IL19

IL22RA1

AGTR1

ABCB1

JAK1

TF

MAP3K8

FCGR2B

HTR2A

BMP1

PRF1

GNS

GAL

CD274

SNAI1

TNFRSF17

FGF7

P2RX3

SRF

PRKN

SHBG

LECT2

IL26

KIR2DS4

FGA

MBL2

SMPD1

LBR

F5

IGF2R

GZMB

FGF3

GDF6

CD34

EPO

DDB1

SP7

SIGLEC1

PRRC2A

GGT3P

MDM2

KIT

NR1H4

LRP2

CDK1

SERPINF1

PF4

TNIP1

ARL13B

MIR126

EGFR

CDK2

HNF4A

CYLD

IRAK3

EIF2AK3

LMNB1

FCGR2A

HELLS

WRN

ESD

CPB2

SERPINF2

CHIA

BST1

BRD3

FLG

LTB

CTRL

NPPC

HLA-DRB5

GPBAR1

BTLA

IFNA1

CASC3

MIR186

CDK6

BTK

FLT3

ABL1

ACVR1

FGFR4

HDAC6

CCND2

MME

GRM1

DBH

PPIB

ROCK1

FLNA

BUB1B

RIPK1

GNAQ

PBX1

PRKDC

RPS6KB1

CD19

KCNQ1

CBS

F10

ASS1

MVK

GRIN2D

IKBKG

PAH

PRLR

SIRT2

SIRT3

GHSR

GJB2

GK

FAH

AK1

MCL1

CCND3

HLA-DRA

DIABLO

MUC1

GPX4

EIF4EBP1

SH2D1A

NPC1

PDE6A

PRNP

SLC6A4

NPR2

SSTR2

TAP1

TLR1

OTC

HTR1A

KCNJ1

USP7

GNAI3

BIRC3

ACTN4

MTR

CD8A

PAX2

PAX5

PSMB9

ILK

TNFRSF13B

LFNG

TK1

PRPS1

WNT1

CACNA1F

APEX1

F11

ATP7A

ATIC

FDPS

ERCC1

ERN1

BCL6

BIRC2

ADAMTS13

CDK9

HEXA

DHODH

GRIK1

CD22

CD27

RFC1

EMD

NPHS1

HTR3A

PPP1CB

PPP1CC

PRDM1

PKD1

PLOD3

SIRT5

SLC19A1

SSTR3

LAMA1

TUBB4A

RAN

STEAP3

STAT5A

TRPM6

XPO1

XPA

GFM1

CARTPT

CBX5

ARSB

ANK3

BCL2L2

MCOLN1

ACO1

ADH4

FRK

CKM

CLCNKB

GPNMB

COL4A3

SGSH

SEC24D

IGFALS

PLA2G10

SLC9A3R1

PLCE1

SLC22A12

TNFRSF9

TMPRSS6

LAMP2

LIG3

STS

TAP2

TAT

PSMB5

TP53BP1

RPE65

PSMB10

TFR2

XRCC5

ATN1

M6PR

MBTPS2

ADH1B

GALC

APBB1

LOXL1

ERAP1

CLCN4

ROBO3

CD226

CD2AP

CD33

COL4A4

SPG7

SI

SKP1

PAX1

SLC29A3

OCA2

PSMD14

SPTA1

SSTR1

TRHR

IRF4

RBBP4

TAPBP

SUMF1

MTRR

NAGLU

NARS2

SUV39H1

TFE3

VAV3

LMNB2

CCS

BANF1

MEOX1

ADH1A

ACTC1

BRDT

CLCN5

COMMD1

MOCOS

HPGDS

RPS26

SLC31A1

TNFRSF8

UCHL5

TRH

RBX1

TMEM38B

CD1D

STX16

GDF3

GCM2

GNRH1

GNA15

MICB

LOXL3

AP2S1

ASAH2

AFF2

FOXC1

FCAR

ERCC8

CD5

GSTZ1

CNN1

CKS1B

CLDN16

CHST14

HCCS

GPR143

MPG

NEDD8

RPL34

HLA-DMB

IKZF3

NPHS2

ICMT

SNX10

PCLO

PNPLA3

PLEKHM1

PLS3

SIRT7

SLC24A5

SLAMF7

RPS3A

SSTR4

SSTR5

TWIST2

U2AF1

LEMD3

SYNE1

SUCLG2

TKTL1

UBA7

CAPZB

GHRH

ATOX1

FKBP10

BSND

CYBRD1

CLN8

DNAJB11

DIS3

MOCS1

CD69

HLA-DMA

HLA-DOA

CRTAP

NPSR1

PROP1

PLEK

SIRT4

SLC45A2

ONECUT1

NUP153

SLAMF1

PIGT

KLRC1

KLRK1

INF2

KDM4C

CARD8

GNPTG

CCRL2

ALX1

ALX4

FUZ

FRMD7

HPS4

HEPH

CELSR3

HGSNAT

MLANA

MYOM2

GPRC6A

DDX39B

NCR2

NCR3

CPNE3

COPE

PDLIM5

PRSS2

NIT2

PMAIP1

PIPOX

LEMD2

SYNE2

TBX6

LDB1

NAT10

KRT86

LRRK1

ASAP2

COL15A1

MYO18B

DBP

NYX

KIAA0319L

LDB2

OSTM1

RCE1

ZNF23

FXYD1

CABP4

CLEC16A

HES7

RIPPLY2

CFHR2

NAA50

GPS1

DAZAP2

SPECC1L

SIAE

PAPPA2

IGDCC3

PACSIN3

IFT43

SCFD1

NT5C1B

PGGT1B

LCOR

TSR2

TAGAP

SUN1

SUN2

DNAH8

NAA30

SAMM50

TGDS

ZNF354A

VSX1

GATB

GBA3

MAGEA3

FBN3

EPDR1

BLOC1S1

COG1

PPP1R12C

SAA2

PLB1

LIN54

TTI2

TBL1X

SUPT20H

P3H2

THSD4

ZNF346

ZSWIM6

VIT

FAM135A

MESP2

MBD6

ADCK2

GPR180

CEP350

HJV

H2AX

IFITM5

ONECUT2

SHROOM2

SSPN

LACC1

SRCIN1

ZNF334

ZNF664

YIPF3

F8A1

ATAT1

ALX3

EPGN

PDF

TMEM222

NARS1

ZNF469

FAM102A

MAGEC1

CNTD1

LAMP5

LARP6

KIAA0895

ADIRF

CARS1

FAM189B

ANKAR

CTAG1B

MT-CO1

OR2D2

OFCC1

SYNE4

P3H4

RBM33

IGLL5

STH

MT-CO3

LRMDA

CCDC154

RIPPLY1

TMSB15A

CTAG1A

TENT5C

PLPPR3

KRTAP4-11

KCNJ18

DIRC1

FRG2C

LEXM

NEMP2

TP73-AS1

MIR125A

MIR135A1

MIRLET7B

MIR196A1

MIR342

MIR28

FBXL19-AS1

MIR331

MIR99B

SNHG28

SNHG29

MIR485

RNU105C

PCAT1

MIR561

SCARNA22

MIR363

ST3GAL6-AS1

CCAT1

HCG14

LAMA5-AS1

PDIA3P1

MIR4497

LOC100130744

IGHV4-38-2

GPR143P

CNC2

OASD

OA3

LOC106050102

LOC106050103

LOC106146143

LOC106146144

LOC106146150

LOC106146152

LOC106627981

LOC106627982

GSR

HDAC9

PTN

PARP1

PRKCA

ASTN2

CD68

MUS81

SPCS1

HAS2

PLA2G3

PLAT

IGFBP5

ITGB1

CREB1

LRP6

ADAMTS1

IL21

UCN

XDH

IGFBP7

IL1RAP

TH

PECAM1

IL1R2

IL9

DEAF1

GPR22

CAV1

CXCR2

ATF2

HRH2

PDPN

ACVRL1

IL1RL2

CXCR1

TYMP

SERPINB2

XIST

FOXD2-AS1

DPP4

MAP2K4

LUM

MCAM

SLPI

HMMR

GZMA

TCF7L1

CHI3L2

BMPR1A

BIRC5

ADIPOR2

CCL8

CLEC4A

ADAMTS8

ALPP

MAP2K6

PRELP

CHAD

KNG1

HBEGF

SOD3

MMP28

MIR320C1

NOS1

NOD1

AQP9

MAP1LC3A

ETS2

STAB1

SCUBE1

NLRP12

PAPSS2

LOC111365141

L3MBTL2

L13304-025

AB372731

DUSP1

S100B

NGFR

MMP16

ARNTL

CCL22

MIR30C1

B3GAT3

CST3

HMGA2

SFRP1

HMGB2

CABIN1

IL1RL1

HYAL2

MRC2

CYTL1

THRA

ATF6

HMGCR

UTS2

USP33

COL6A4P2

MAP3K5

PPARGC1A

CCR4

SERPINB5

PTGES3

PPBP

SP3

SERPINA4

SERPINE2

TFF3

IL18RAP

PITRM1

IFNL1

PTGER2

SIK3

MIR4435-2

COL27A1

ITIH1

ILF3

CEACAM4

DSP

ADM

CSTA

FOXD1

AHR

YY1

GALE

SLC17A5

WNT16

CHRDL2

DCSTAMP

NR1H3

IL12A

C5AR1

SLC23A2

GSTM1

SCG2

MIR29B2

MIR29C

MIR338

BMPR2

ULK1

XYLT2

CS

PTH2R

DNTT

MIR23A

HOTAIRM1

OAP

LOC106728418

AR

PPP3CA

ESRRB

GSTP1

CYP2D6

FAAH

CD3D

PRKAA1

ENO2

PIK3CB

BECN1

C5

HCFC1

MECP2

HYAL1

GANAB

GPX3

DDIT4

PFKFB3

SHMT2

PDE5A

NTN1

PTPRD

SULT2A1

PRKAR2B

PDE4A

TFF1

HES1

NCOR2

CSPG4

PDGFC

PRDX4

PTGES2

TAGLN

CBX4

APOL1

EIF4G2

CCL7

LPA

CRLF1

CHKA

NOX1

SDCBP

SMN1

KLK11

TFF2

BOK

ABCD2

FUT1

HS6ST2

HGFAC

DGCR8

DPP7

IL17RC

CD160

DUSP19

DSTN

OXA1L

CCL16

FOXP4

CREB3

EN1

CRTAC1

CKLF

NID2

SUB1

CCL14

BCAP29

NKX3-2

CCL15

MICAL3

SHROOM1

AKNA

FNDC5

MIR196A2

MIR152

MIR138-2

MIR200B

MIR23B

MIR211

MIR301A

MIR451A

MIR1246

MIR1277

MIR634

TP53COR1

LOC112543491

HPBP

LOC110283621

MAPT

USP8

ABT1

MIR499A

CEACAM8

CEBPB

MAPK10

TNFRSF6B

WNT7A

SETD2

LDHB

SETD1A

MIR146B

BHD

PCSK9

NPM1

CYP11A1

NF1

SLC25A13

GPD2

KAT5

AP3B1

TBX2

HERC2

ADD1

ATP2B1

GAK

CRADD

CDC7

PAX7

MMP24

GRK4

EIF2B1

HTT

SOCS2

SLC39A4

LCT

TACC3

USP24

ANXA3

DPEP1

NFAT5

SLC30A10

ITIH4

LTBP3

MCAT

PAPPA

NISCH

SMYD3

SPTBN2

JPH3

RASGRP3

TBX4

PUF60

CYP2A13

SCYL1

PTPRG

ABCB9

MAT2B

GALNT5

CACNG5

B9D2

FAR2

MAGI1

AAGAB

CEACAM3

CEP250

RNF4

NEK4

EIF6

HLA-DPA1

CPNE1

SMG6

PRDM2

SCRIB

SART1

SLC44A2

POLA2

STRA6

DYNC1I1

SH3BP2

YKT6

GGT7

AGAP1

BMP2K

CPSF6

BTN3A1

RFX3

RPGRIP1L

DDX10

NCAM2

PLXNC1

TCTN2

RAD54L2

TIPIN

NCOA6

RNASEH2C

KCNIP4

EYA2

ACER3

CDK2AP1

RFT1

RPL29

SF1

TNIP2

SF3B2

ZMYND8

FRMD4A

LRIG3

EXOSC4

FAM136A

LYPLAL1

ANKRD6

BTN2A1

FOXL1

BTN2A2

CHD9

DLGAP2

HNRNPUL1

PARP10

SENP6

IRX3

TNFSF8

TAS1R2

RBM39

RBM12

SNX32

NDST4

MRPL11

LRRN1

ATF7IP

ERGIC3

ERP27

ABHD14B

BTN3A2

COLGALT2

NELFA

SF3A2

PPM1H

SBNO1

RPRD2

POGK

ISLR

RAPH1

KIAA1217

KNTC1

KIF26B

RBM6

TMEM91

LMAN1L

FSTL5

CCDC91

APOBEC4

ANAPC4

BBX

AAR2

BTN3A3

MON2

DDX55

MOB3B

RWDD2B

RRP9

NT5DC2

ISLR2

TTLL12

TSKU

KIF12

ZFYVE28

ZCCHC8

ZWILCH

UVSSA

VPS51

FILIP1

EPPK1

C11orf80

HECW1

CCDC33

MPHOSPH9

NRBP2

SRBD1

TENM2

THSD7B

GALNT18

CCDC62

H1-4

HMGN4

MLXIP

HAUS3

EIF1AD

SPATC1

IQCH

IZUMO4

KAZN

ZFPL1

ZC3H3

FAM53A

LURAP1L

FAM83C

BRINP1

BTNL8

GSDMC

CEACAM21

H1-2

CCDC97

RSRC2

NKAIN1

POLR2M

KIAA1755

KLHL42

ZBED5

FAM193A

FAM98A

H3C3

CCDC54

TMEM241

LSMEM1

C12orf60

EQTN

H1-3

TMEM129

LRRC43

CNBD2

H2BC4

H2BC9

C11orf87

H2AC6

TMEM61

ZNF345

ZNRD2

C8orf82

ATP23

CSNK2A3

PLPP6

SMIM4

CCDC26

C16orf95

MS4A13

PCNX3

MANSC4

DELEC1

RFLNA

STIMATE

MINAR1

BTN2A3P

LINC02875

SMIM23

LINC01006

DNAH10OS

LINC00114

NOP14-AS1

GDF5-AS1

CTBP1-DT

MIR661

HCG11

GUSBP2

LINC01088

FAM83C-AS1

HCG23

NR2F2-AS1

SNORD19B

SNORD16

IQCH-AS1

SAMMSON

ZC3H11B

LINC00977

MMP24OS

ILF3-DT

LINC01094

LINC01507

MIR1289-1

GARS1-DT

LYPLAL1-AS1

ATP2B1-AS1

ASTN2-AS1

MDFIC2

DUBR

LINC01574

LOC101928731

ENSG00000255224

ROCR

ZNRD2-AS1

LSP1P3

LOC101928540

SBNO1-AS1

KIF26B-AS1

ZNF664-RFLNA

LINC01754

LINC01338

LINC02399

HOMER3-AS1

MPPED2-AS1

RPL32P1

ENSG00000243224

ENSG00000237174

RPL35AP

ENSG00000256546

ENSG00000255730

ENSG00000249592

ENSG00000254461

ENSG00000255320

ENSG00000255038

ENSG00000254855

ENSG00000254632

ENSG00000245156

DUS4L-BCAP29

ENSG00000253926

ENSG00000261384

ENSG00000261324

ENSG00000267122

LINC02234

RNA5SP301

MTND4P14

NPM1P14

ENSG00000227554

ENSG00000234352

ENSG00000234091

ENSG00000231612

ENSG00000254094

ENSG00000258216

ENSG00000248538

ENSG00000228322

ENSG00000270195

ENSG00000272462

ENSG00000261578

LINC02742

LINC02650

H2AC5P

RPL26P20

SOD2P1

ENSG00000225884

PPIAP1

ENSG00000237530

ENSG00000238224

ENSG00000223727

ENSG00000225050

ENSG00000207009

ENSG00000207189

ENSG00000200998

ENSG00000234892

LOC105373944

ENSG00000248112

ENSG00000257042

TPT1P9

ENSG00000250098

ENSG00000250453

ENSG00000254614

ENSG00000241956

RNA5SP349

RNA5SP355

ENSG00000275443

ENSG00000260773

LINC02752

ENSG00000282246

RF00017-5093

ENSG00000287373

ENSG00000286177

ENSG00000285108

lnc-TNIP2-1

CNN2P10

MRPS33P3

lnc-GLIS3-2

LOC101928268

CSP2

NPAP1P4

ENSG00000238156

RPL21P34

lnc-KCNK7-4

ENSG00000173727

ENSG00000279093

ENSG00000273179

ENSG00000273771

LINC02680

ENSG00000272588

lnc-TIMM29-1

MARK2P15

RF00017-7373

RF00017-7375

lnc-CD47-7

piR-40096

ENSG00000288096

lnc-BRD2-2

lnc-BRD2-3

piR-39858-250

ENSG00000287632

RF00017-5095

lnc-SSSCA1-1

FJ601676

lnc-SOCS2-6

ENSG00000285144

ENSG00000285577

ENSG00000285914

ENSG00000286910

lnc-APOBEC4-5

piR-31751

lnc-B9D2-2

lnc-CTDSP2-7

lnc-TACC3-2

lnc-STAB1-1

RF00017-7406

lnc-ZMYND8-10

piR-34822-068

lnc-YKT6-2

lnc-ETS2-8

piR-42694-043

lnc-DYNC1I1-4

piR-43098

lnc-TTLL12-1

piR-50208

lnc-IQCH-7

lnc-FAM83C-2

lnc-FAM83C-3

HSALNG0123133

ENSG00000238232

ENSG00000222658

NONHSAG011935.2

ENSG00000219682

lnc-KIF12-2

lnc-KIF12-3

SNODB718

LOC107987014

RF00017-2809

lnc-PRPF18-2

ENSG00000251446

lnc-PLEC-4

L13304-005

ENSG00000239793

piR-50446

lnc-NCOA3-18

ENSG00000277135

ENSG00000277749

ENSG00000277829

ENSG00000273254

ENSG00000264997

lnc-ADD1-6

lnc-ADD2-2

ENSG00000280904

lnc-SMYD3-5

piR-32559-090

lnc-RPGRIP1L-2

RF00017-596

5MWI_A-023

ENSG00000283271

ENSG00000283517

RF00017-3161

lnc-COLGALT2-6

lnc-GGCT-3

piR-43105-319

H3P27

lnc-HFM1-3

MS4A19P

LOC100288365

RPL14P6

HSALNG0059706

HSALNG0026174

MN308750

LOC105372401

lnc-KLHL42-2

NONHSAG001562.2

piR-36756-038

SMC4P1

lnc-NT5DC2-1

LOC107986989

ENSG00000256378

lnc-PBRM1-2

RF00017-1169

piR-43107-151

piR-57133-366

piR-57137-006

piR-49204-003

RF00017-5583

lnc-BRD2-4

piR-39858-279

piR-56497-029

FJ601684-189

lnc-ARHGDIB-2

ENSG00000286953

piR-56368

AB372616

piR-32023-046

ENSG00000286417

MF281437-018

lnc-TACC3-3

lnc-TNC-7

HG984098

MN297574

lnc-EFCAB2-7

piR-60429

piR-52471-007

RF00100-076

RF00951-040

HSALNG0117900

lnc-NEK4-1

piR-46368

LOC105369890

LOC101929770

piR-62029

lnc-NACA2-9

piR-61945-087

LOC105373943

ENSG00000244712

RF00001-077

LOC107986000

TRUND-NNN9-1

piR-30534

lnc-LYPLAL1-11

NONHSAG012622.2

RF00004-011

ENSG00000278892

ENSG00000279304

ENSG00000274075

lnc-ADAM10-10

piR-56497-036

LOC105377720

LOC105377721

LOC107986600

LOC107985895

ENSG00000259493

ITGAV

SOCS3

TSLP

NTRK1

ATG7

MIR136

KLF3-AS1

ANG

GC

JUNB

CD55

CXCL16

ALOX5AP

TNFSF12

CPOX

PDGFRA

MAP3K7

MAP2K3

ANXA2

F2R

CD151

BMP3

CAPN2

NRP1

FST

HSD11B2

E2F2

AMBP

GADD45B

FZD1

GUSB

LTF

IL24

CD58

IL20

FAM215A

PDGFB

CD59

DDIT3

CFP

ICAM3

LGALS3BP

POMGNT2

MYDGF

PROC

HDC

ITGA1

PTX3

HAS3

SOX4

RNASE3

CALR

TERT

MAPK9

GSN

FURIN

RHOA

ICAM2

MAP3K2

ANGPT2

S1PR1

TICAM1

IGFBP4

SMAD7

FCGR1A

BPI

IL17B

ACKR3

AIF1

ADGRE5

MIR148A

TXN

LAIR1

CXCR6

F2RL2

C4BPA

TYK2

ITGA2B

STAT6

MAP3K1

ITGA5

ENTPD1

CD9

TNFRSF12A

DKK2

SLC3A2

THPO

CD63

CHRD

CD84

SMAD5

KERA

IL36A

TSPAN4

DEFB103B

PCNA

ABCA1

MMP19

VEGFC

PRDX2

TFDP1

ANXA6

E2F1

PRDX3

RAMP2

CCL27

SNTB1

CORIN

PDGFRB

MAP2K1

NTRK2

FLT4

G6PD

MSH6

C3

GFAP

CCNB1

HSPA8

IRS1

HSD3B2

PTGER3

FGF5

ANGPTL4

SEMA4A

COL18A1

POLG

KLF4

CITED2

ELK1

SKIL

ITGB7

PLA2G5

CMA1

NTF3

ID1

THBS4

ADAMTS2

MT2A

LAMA5

SEMA3C

MARCO

HOXD9

HOMER1

AZU1

ADAMTS12

RND1

CALCB

A1BG

IFI27

ZBTB38

VSIG4

CPQ

ITGBL1

H2BC21

SELENOS

H3C14

MIR194-1

PIK3CD

CSNK2A1

SLC9A1

HSPB1

ZAP70

BMPR1B

MYB

PRKCH

RARB

ETS1

GRIN1

RRM2B

ITGA6

STK11

ALDOA

CYP3A4

EEF2

CDH11

HPRT1

SCNN1B

SLC2A3

REN

GDNF

CAMK2G

MAP3K3

ADAM9

EPHX1

CYBB

EPHB1

DDX58

SLC9A3

IL7R

TTN

TYRO3

LEF1

TFAP2A

EFNB1

GFPT1

MAP3K11

CD46

HMGA1

MYCN

PTPN2

SUMO1

DAG1

BAP1

AICDA

C1QA

MMP15

GRK6

IDO1

SCARB1

PTPRB

IRF8

KCNA2

RAG1

ITCH

THBS2

VIPR1

C1QB

C1QC

CCNA2

FABP4

LTB4R

AGRN

FZD10

CENPE

PDGFA

RXFP1

SMAD1

HSF1

PLXNB1

TPH1

LAMA4

SREBF1

ID2

B3GAT1

BATF

MAP3K4

AGTR2

ACD

GAB2

FOXO4

FSCN1

GSTA4

CPA6

HDAC10

DHRS3

EEF1A1

CD209

SIRPA

NRP2

NR2C2

INHA

PHOX2B

TNFRSF14

TRAP1

TNFRSF10D

RAMP1

TIRAP

P2RX1

PDYN

ITGA9

TIE1

LPIN2

ALCAM

GRPR

CDKN3

CDO1

CDH13

PADI3

IFI16

PRMT5

TNFRSF18

TREX1

RAG2

LTB4R2

ATG12

LTBR

BCL3

CAMP

CSGALNACT2

IL15RA

PCM1

SEMA3F

TAB3

ID3

CCR9

G3BP1

CAPZA1

CHRDL1

EDIL3

SLC9A2

TRAIP

TPST1

TRIM2

KIR3DL1

TEP1

TERF2

RCOR1

OXT

BHLHE41

ADAM28

HPX

CD5L

MKKS

DEFB1

TINAGL1

TNFRSF10C

TTF2

RASGRP4

KIR2DL4

IVNS1ABP

DEFA5

IL17D

PDIA2

IER3

OSCAR

SRGN

TNFSF18

C1D

BAIAP2L1

ATP6V0E2

ADPRH

ADGRE2

CLEC4E

DEFA6

SAP30BP

PIWIL2

PIWIL4

UCN3

KIR3DL2

SPA17

MYCL

XCL1

VASH1

DEFA3

SNX19

KIR2DL1

KIR2DL3

KIR3DL3

PGPEP1

ZNF277

GALNT15

GCNT4

LVRN

HECTD2

PCOLCE2

TTC38

PTPA

LBH

EFS

CLEC5A

ADM2

FNDC1

FAM122B

UCN2

ZNF302

H3C1

SFMBT2

SAAL1

CNMD

TERC

RBIS

KIR2DS2

KIR2DS3

KIR2DL2

MIR143

KIR2DL5A

KIR2DS1

MIR124-1

KIR3DS1

MIR20A

MIR216A

MIR19B1

MIR608

MIR519B

MIR92A2

MIR558

MIR602

KIR2DP1

TALDO1P1

LOC108021846

PLA2G6

MMP11

SELENOP

CASP7

CSNK1D

IFNGR1

FSHR

MAPKAPK2

GPX1

IDE

TPO

TRPC3

RDX

TRPC6

GNB3

FGF10

MC2R

MSN

DLG4

CSNK2B

PIK3C2A

ITGB6

RBPJ

AMPD2

DCC

SLC20A2

TXNRD2

KLK3

EZR

ADORA2A

HRH1

SMURF1

OPRL1

ITGA2

HSPG2

P2RY2

FGF14

HGS

ENPP2

SNCAIP

SLC20A1

ITGAX

REST

LBP

CBX3

CD70

PAPSS1

MC5R

COPS5

ITGA11

CENPJ

MIP

FGF13

FOSB

FOSL2

BRD1

PTGDR2

TRPC5

APOM

CD276

GABPA

RHOD

SFRP5

SERPINA7

KCNN1

ITGA10

ACKR1

MRPL17

DPT

NOX5

P4HTM

NUPR1

ABHD16A

GPANK1

C6orf47

CGAS

CIP2A

LY6G5C

HOTTIP

MIR770

FRA1H

FADD

KITLG

ERRFI1

CYP2C9

HNRNPA2B1

CALCRL

ELP1

MAP3K4-AS1

MAP2K2

NR1H2

SQSTM1

KRT18

PRKAB1

PTGIS

TXNRD1

LIFR

GNAI1

F13A1

A2M

CRHR1

TBX5

EIF2S1

SNAI2

ALPI

SERPINA6

CNTF

IL21R

DIO1

GAST

AXL

ATR

CDC42

NTRK3

JAK3

PGR

IRAK1

PLK1

CASP10

MAPK13

AXIN2

MEF2C

RHEB

IL2RG

PRMT1

EGLN1

PLD1

IRAK4

VCP

YWHAZ

GLI1

DDR1

IFIH1

PITX2

PNLIP

ODC1

PTGIR

PIN1

IRF3

PTK2

ATF4

CLTC

NR4A1

SDHA

KDM6A

TLR7

PDE8B

WEE1

CSF2RA

S1PR2

SMAD9

TNFSF15

RASGRP2

EIF2AK2

TGFBI

GP6

MAP3K14

CLEC7A

CBFB

CISH

GAS6

IL10RB

NTF4

NPPB

UGT2B7

SRD5A1

PDE4B

EIF2AK4

DLX5

ABCB7

AREG

AMD1

AIMP1

AGRP

MDM4

RGS4

RGS10

COL4A5

CYSLTR1

PCSK7

ENPP3

PTPRK

TRADD

TRAF3IP2

UGT1A9

TRIM32

ROM1

HSPA1A

PLAA

ANXA7

BNIP3

HNRNPD

SEMA7A

PYY

PDIA4

GLRA3

HNRNPC

H6PD

DSE

RASGRF1

LIPF

RGS5

RGS7

HCRT

EHF

HSPB2

IAPP

UXS1

LMOD1

MAPRE3

EBI3

CD83

NELL1

IL1F10

PHOSPHO1

LAMP3

TXNDC5

TSPAN9

SPAM1

ITGAE

CCL17

AP2A1

ADAMTS16

UGT1A3

TET1

EBAG9

MADCAM1

CHST4

PIEZO1

LAIR2

IRX1

ARL15

HIVEP3

TNIP3

LILRA5

TP53AIP1

RNASE7

CNN3

RNPC3

ENGASE

COL20A1

PPARGC1B

IL36B

CSN1S1

JPH4

VEGFD

GCFC2

CD24

GDPD4

CCN5

CSRNP1

ERV3-1

HEXD

NAA80

TSBP1

MIR192

MIRLET7A1

MIR155HG

MIR191

MIR181B1

MIR302D

MIR302B

HAS2-AS1

SAA3P

GRASLND

UGT1A

TRAP

STQTL2

EDM5

HOA

OS2

SEMDBCD

FRZB1

SRFP3

OS1

GOA1

PLAP1

OS3

PLAP1

AGC1

CSPG1

MSK16

SEDK

SSOAOD

ACAN

AGC1

CDMP1

SYNS2

OS5

BDA1C

SYM1B

DUPANS

CDMP1

SYM1B

SYNS2

OS5

BDA1C

CDMP1

SYM1B

5-LOX

MMP-2

TrkA

COX-1

FXR

MAPK12

COX-2

COX

MMP-13

IKKB

BDKRB1

MMP-3

NaC

p38

MMP

ALOX

Wnt pathway
